# A novel monoclonal antibody targeting carboxymethyllysine, an advanced glycation end product in atherosclerosis and pancreatic cancer

**DOI:** 10.1371/journal.pone.0191872

**Published:** 2018-02-08

**Authors:** Ulrika Wendel, Nina Persson, Christian Risinger, Eva Bengtsson, Björn Nodin, Lena Danielsson, Charlotte Welinder, Gunilla Nordin Fredrikson, Bo Jansson, Ola Blixt

**Affiliations:** 1 Chemical Glyco-Biology Laboratory, Department of Chemistry, Faculty of Science, University of Copenhagen, Copenhagen, Denmark; 2 Department of Clinical Sciences Malmö, Scania University Hospital, Malmö Lund University, Malmö, Sweden; 3 Division of Oncology and Pathology, Department of Clinical Sciences Lund, Lund University, Lund, Sweden; 4 Clinical Chemistry and Pharmacology, Department of Laboratory Medicine, Lund University, Lund, Sweden; 5 Centre of Excellence in Biological and Medical Mass Spectrometry “CEBMMS”, Biomedical Centre D13, Lund University, Lund, Sweden; Iowa State University, UNITED STATES

## Abstract

Advanced glycation end products are formed by non-enzymatic reactions between proteins and carbohydrates, causing irreversible lysine and arginine alterations that severely affect protein structure and function. The resulting modifications induce inflammation by binding to scavenger receptors. An increase in advanced glycation end products is observed in a number of diseases e.g. atherosclerosis and cancer. Since advanced glycation end products also are present in healthy individuals, their detection and quantification are of great importance for usage as potential biomarkers. Current methods for advanced glycation end product detection are though limited and solely measure total glycation. This study describes a new epitope-mapped single chain variable fragment, D1-B2, against carboxymethyllysine, produced from a phage library that was constructed from mouse immunizations. The phage library was selected against advanced glycation end product targets using a phage display platform. Characterization of its binding pattern was performed using large synthetic glycated peptide and protein libraries displayed on microarray slides. D1-B2 showed a preference for an aspartic acid, three positions N-terminally from a carboxymethyllysine residue and also bound to a broad collection of glycated proteins. Positive immunohistochemical staining of mouse atherosclerotic plaques and of a tissue microarray of human pancreatic tumors confirmed the usability of the new scFv for advanced glycation end product detection in tissues. This study demonstrates a promising methodology for high-throughput generation of epitope-mapped monoclonal antibodies against AGE.

## Introduction

The non-enzymatic reactions between protein primary amines and carbonyl carbons [1] leading to advanced glycation end products (AGE) were first studied by Louis Camille Maillard [2]. The reaction is initiated by nucleophilic attack by a protein amine towards the carbonyl carbon of a carbohydrate. The resulting reversible Schiff base formation can then rearrange to the more stable Amadori adduct. If the reaction proceeds, the products become more complicated to predict [3]. Degradation of the attached carbohydrate chain, under the influence of radicals, results in a large variety of irreversible structures. The affected sites are mostly the amines of lysine and arginine residues and their altered confirmations and charges cause major structural and functional changes in proteins [4,5].

Enzymatic defense against AGE modifications is present in vivo through the activity of glyoxalases [6], but it does not always manage to reverse the glycations. Thus, AGEs accumulate with time, especially in long-lived proteins [7–9]. This accumulation leads to stiffness of tendons and increased systolic blood pressure, which are common symptoms of aging [10].

In addition to their role in natural aging, AGEs have been observed in diseases such as atherosclerosis [11], rheumatoid arthritis [12], Alzheimer’s disease [13,14], diabetes [15,16], and various forms of cancer [17,18]. AGE-modified proteins are recognized by several cell-associated and circulating scavenger receptors, such as the ubiquitous receptor for AGE (RAGE). RAGE has the potential of activating inflammation through the NFκB pathway [19] and increases in the expression of RAGE are observed in many diseases [20]. AGEs are also known to induce oxidative stress and immune responses [19,21,22].

Because of the unpredictable nature of this non-enzymatic glycation process, it is important to develop specific reagents and tools that can detect specific sites of glycation [23]. Most assays for AGE detection give information on the average total content of glycated adducts in a biological sample instead of glycation of specific sites and are also associated with high inconsistency between different measuring times and between types of methods [24]. Since glycation also occurs in healthy individuals, it is of the outmost importance to separate and detect the epitopes that relate to states of disease. Differences in glycated sites between diseased and healthy individuals have been highlighted in a number of recent studies. Frolov *et al*. detected differences in the Amadori formation patterns between human serum albumin (HSA) from healthy individuals and from individuals diagnosed with diabetes, highlighting the importance of detection of specific glycation sites [23]. The same result was obtained by Zhang *et al*. in an earlier study [25].

In addition, there is a need of measuring advanced glycation and not the early Amadori adducts, since the latter does not enable more detailed analysis of complications caused by a high and fluctuating blood sugar concentration [23,25,26].

Monoclonal antibodies have the potential of detecting and quantifying single glycated epitopes, but the ones currently available do not have described epitopes, disabling targeted detection of specific AGE-modified sites [24,27]. Thus, there is a large need for monoclonal antibodies with a well-defined binding-pattern, to fill the specificity gap and make a base for an improved analytical capacity.

This study aims to present a methodology for generation of epitope-mapped anti-AGE monoclonal antibodies using a phage-display platform and parallel microarray analysis against AGE-target libraries.

## Materials and methods

### Reagents and chemicals

Proteins, carbohydrates, and other chemicals were purchased from Sigma Aldrich (St. Louis, Missouri, USA) if not stated otherwise. SPPS building blocks and chemicals were purchased from Iris Biotech, GmBH. Nexterion^®^ Slides H, NHS (SCHOTT, AZ, USA), BI201 human IgG1 was a gift from Bioinvent International AB, Lund, Sweden. D-glucose (Amresco, OH, USA), glyoxylic acid (Merck, Darmstadt, Germany), pyruvic acid (Acros Organics, Geel, Belgium), TentaGel S RAM resin (Rapp Polymere, Tuebingen, Germany), *Escherichia*. *coli* XL1-Blue bacteria (Agilent, CA, USA), Ni-NTA agarose (Qiagen, Copenhagen, Denmark), Mini-PROTEAN^®^TGX^™^ 4–15% Precast gels (BioRad, Copenhagen, Denmark), Micro BCA^™^ Protein Assay Kit (Thermo Scientific, MA, USA), OCT/Tissue-Tek (Qiagen, Copenhagen, Denmark), VECTASTAIN ABC HRP Kit (Vector Laboratories, BioNordika, Herlev, Denmark), ImmPACT DAB Peroxidase (HRP) Substrate (Vector Laboratories, BioNordika, Herlev, Denmark), RNAlater^®^ solution (Qiagen, Copenhagen, Denmark), RNeasy Plus Mini kit (Qiagen, Copenhagen, Denmark), ThermoScript Reverse Transcriptase (Invitrogen, Carlsbad, CA, USA), immunotubes (Thermo Scientific, MA, USA), magnetic streptavidin beads M280 (Invitrogen, Carlsbad, CA, USA), aprotinin (Roche, Basel, Switzerland), SfiI enzyme (New England Biolabs, Ipswich, Massachusetts, USA), Histochoice (Amresco, Solon, OH, USA).

### Synthesis of glycated proteins and peptides

Solutions of 50 mg/mL BSA, 50 mg/mL HSA, 10 mg/mL fibrinogen from human plasma, 0.35 mg/mL collagen from human placenta and 10 mg/mL BI201 human IgG1 in PBS were each incubated with 0.5 M D-glucose/0.5 M D-(-)-fructose/0.5 M D-(-)-ribose, 0.1 M glyoxal/0.1 M methylglyoxal/0.1 M DL-glyceraldehyde in varied sets of parameters (temperature, 37°C/50°C; pH, 7.2/10; time, one day/two days/three days/one week/two weeks/three weeks/four weeks). CML-modifications were accomplished by reductive amination in 150 mM NaCNBH_3_ with 45 mM glyoxylic acid in 37°C for 1 day. Carboxyethyllysine (CEL)-modifications were produced in the same manner as CML, but with 45 mM pyruvic acid instead of glyoxylic acid. Reactions were disrupted by either snap freezing to -80°C or dialysis to remove reactive carbohydrates.

Peptides were synthesized as earlier described by Risinger *et al*. [28] on tip-supported beads (1.4 μmol scale), using automated 9-fluorenylmethoxycarbonyl (Fmoc) SPPS on a SyroI (Biotage, Uppsala, Sweden) with the software SyroXP. Two equivalents of Fmoc-L-CML(oBut)(Boc)-OH was used together with four equivalents of 20 standard Fmoc-protected amino acid building blocks and Fmoc-O2Oc-OH spacer and coupled onto TentaGel S RAM resin (0.24 mmol/g loading density) using standard SPPS solvents. Peptide sequences were secured by strategic capping with acetic anhydride before Fmoc-deprotection. Succinylation and biotinylation were performed by selective Aloc-deprotection of Aloc-L-lysine(Fmoc)-OH under argon flow, using borane dimethylamine complex and Tetrakis (triphenylphosphine)palladium(0), then coupling of D-biotin or mono-*tert*-butyl-succinate, before Fmoc-deprotection. Resulting peptides were cleaved from the resin with 95% TFA, which was then evaporated with a speedvac Concentrator (Savant, SPD131DDA, Thermo Scientific, MA, USA). Dried peptides were dissolved in 50% MeOH in H_2_O.

### Microarray printing of peptides and proteins

Microarray printing was performed as earlier described [28–31] on hydrogel coated Nexterion^®^ Slides H, NHS using a MicroGrid array printer (60 nL/deposit, quilled pins, 250 μm pitch, BioRobotics, Genomics Solutions, Cambridgeshire, UK) with TAS Application suite software. In brief, glycated peptides and proteins were dissolved in printing buffer (17 mM NaH_2_PO_4_×H_2_O, 133 mM Na_2_HPO_4_×2 H_2_O, 0.03% NaN_3_, pH 8.5) to a final concentration of 700 μM for peptides and 200 μM for proteins to ensure that the microarray spots were saturated with the printed targets. The non-printed surfaces were blocked with 50 mM ethanolamine in 50 mM borate buffer, pH 9.2, before incubations with antibody solutions diluted in PliP buffer (6.5 mM Na_2_HPO_4_, 1.5 mM KH_2_PO_4_, pH 7.4 containing 500mM NaCl, 3 mM KCl, 1% BSA and 1% Triton X-100). Fluorescence signals (relative fluorescence units, RFU) from detected slides were measured using a ScanArray 5000 confocal scanner (PerkinElmer, Massachusetts, USA). Detection antibodies were diluted to 1:500 unless stated otherwise.

### Immunization and antibody library construction

The license for immunization was obtained by Afdeling for Eksperimentel Medicin, University of Copenhagen (license number, 2012-15-2934-00077). Two female 6–7 weeks old BALB/cJBomTac mice were immunized twice with 100 μL 1.75 μg/uL of a collection of AGE-modified proteins in PBS together with Freund’s incomplete adjuvant, (FIA). IgG (10 mg/mL) and BSA (50 mg/mL) had been incubated with 0.5 M D-glucose, 0.1 M glyceraldehyde, 0.1 M methylglyoxal, 0.1 M glyoxal, 0.1 M glyoxylic acid and 0.1 M pyruvic acid for seven days in 37°C except for the glyoxylic and pyruvic acid reactions that were modified for one day. Modified proteins had then been purified from reactants by dialysis, (see Synthesis of glycated proteins and peptides). The second immunization occured 28 days after the first immunization. Mice were sacrificed by decapitation 15 days after the second immunization, after developing an immune response against the injected glycated proteins.

The mouse spleens were transferred to RNAlater^®^ solution and frozen at -20°C until the RNA was extracted with a Fastprep Cell Disrupter FP120 (Q-Biogene) and an RNeasy Plus Mini kit. cDNA was synthesized using 1 μg RNA together with random hexamer primers and ThermoScript Reverse Transcriptase. Specific antibody genes were isolated using PCR and were then cloned into the phage display vector pAK100 (kindly provided by Prof. A. Plückthun, University of Zürich, Switzerland) [32], using the methods described by Schaefer [33]. The outer primers used were 50-phosphorylated to enable an extra rolling-circle amplification step for improvement of the restriction enzyme cutting performance [34]. The phages displaying scFv:s were rescued using VCSM13 helper phages [32]. The RNA from the spleens was isolated, and the antibody-encoding genes were cloned into the phage display vector pAK100 [32,35] used for phage display library construction as earlier described [29,33].

### Selection of AGE-binding scFv:s using phage display technology

Phage display selection was carried out as earlier described by Persson *et al*. [29]. Centrifugation of bacterial solutions was performed at 3000 x g. For culturing of phage-hosting bacteria, LB medium supplemented with 25 μg/mL chloramphenicol and 15 μg/mL tetracycline was used to restrict undesired growth. All phage pool incubations with beads/tubes were performed under rotation/shaking.

Phages were selected against glycated proteins and peptides in three selection tracks, each containing three selection cycles (Table 1). Track A was focused on CML-modified proteins, track B on CEL-modified full-length proteins and track C on peptides containing Fmoc-CML building blocks. Pre-selections with unmodified targets were performed to remove high background binders.

**Table 1:** Preselection and selection targets from three phage display selection tracks, consisting of three selection cycles. Full-length proteins were immobilized onto immunotube surfaces and biotin-coupled peptides were immobilized onto magnetic streptavidin beads.

Selection track	Preselection targets, cycle 1	Selection targets, cycle 1	Preselection targets, cycle 2	Selection targets, cycle 2	Preselection targets, cycle 3	Selection targets, cycle 3
1	Unmodified BSA (immunotubes)	CML-BSA (immunotubes)	Unmodified BSA (immunotubes)	CML-IgG (immunotubes)	Unmodified BSA (immunotubes)	CML-BSA (immunotubes)
2	Unmodified BSA (immunotubes)	CEL-BSA (immunotubes)	Unmodified BSA (immunotubes)	CEL-IgG (immunotubes)	Unmodified BSA (immunotubes)	HSA (immunotubes)
3	Peptide 7 (magnetic beads)	Peptides 1–6 (magnetic beads)	Unmodified BSA (immunotubes)	Glucose-modified HSA (immunotubes)	Peptide 7 (magnetic beads)	Peptides 1–6 (magnetic beads)

See Table 2 for peptide sequences.

Immobilization of preselection and selection targets to immunotubes: Proteins were dissolved in 3 mL in 0.1 M NaCO_3_, pH 9.1, to a concentration of approximately 5 μg/ml and immobilized onto immunotube surfaces for 1h at 37°C. Tubes were then blocked with PBS 0.02% Tween-20, 5% BSA for 1 h at room temperature.

Coupling of preselection and selection targets to magnetic beads: 100 μL of magnetic streptavidin bead slurry was blocked with PBS 0.02% Tween-20, 5% BSA for 1 h at room temperature. Biotinylated peptides, dissolved to approximately 50 nM in 1 mL PBS, 0.02% Tween-20, 3% BSA, were then coupled onto the beads for 1 h at room temperature.

The phage solution was then dissolved 1:10 in PBS, 0.05% Tween-20, 3% BSA and incubated with preselection targets, coupled on beads or immobilized on immunotubes, for 1 h at room temperature. Remaining phage solutions were then transferred to tubes/beads with selection targets and incubated over night at 4°C. Binding phages were eluted with 1 mg/mL trypsin in PBS for 30 min at RT before inhibiting the enzyme with 1/10 volume 2 mg/mL aprotinin.

*E*. *coli* XL1-Blue competent cells in the exponential growth phase were infected with eluted phages for 30 min at 37°C. Infected bacteria were plated on LB agar-plates (1% glucose, 15 μg/mL tetracycline, and 25 μg/mL chloramphenicol) and incubated overnight at 30°C. Resulting bacterial colonies were removed from the plates and resuspended in 10 mL culture medium and concentrated by centrifugation. The bacterial pellets were resuspended in 1.5 mL culture medium with 1/3 50% glycerol for storing in -80°C. A few μL of the produced glycerol stocks were each incubated with 10 mL medium and cultured to OD600 = 0.5 at 37°C. Helper phages (VCSM13, 6 x 10^9^ plaque-forming units (pfu)/mL) were added, after which the solutions were incubated for 30 min at 37°C. Expression was then induced by the addition of isopropyl β-D-1-thiogalactopyranoside (IPTG) to a concentration of 100 μM, followed by incubation overnight at 25°C. Phages were separated the bacteria by centrifugation and precipitated with ¼ volume 20% PEG6000. Precipitated phages were spun down by centrifugation at 4800 x g for 30 min at 4°C.

Resulting phage stocks were titrated and evaluated against 25 targets (Table 2), with or without glycation, printed on an NHS-activated surface (see Microarray printing of peptides and proteins). The selection cycle was repeated twice.

**Table 2:** Microarray-immobilized targets for evaluation of phage display selection tracks.

Target nr.	Target description
1	Peptide 1: biotin-K-O2Oc-O2Oc-A-K*-S-C-O2Oc
2	Peptide 2: biotin-K- O2Oc-O2Oc-C-A-K*-S-O2Oc
3	Peptide 3: biotin-K-O2Oc-O2Oc-P-K*-Y-C-O2Oc
4	Peptide 4: biotin-K-O2Oc-O2Oc-C-P-K*-Y-O2Oc
5	Peptide 5: biotin-K-O2Oc-O2Oc-W-K*-F-C-O2Oc
6	Peptide 6: biotin-K-O2Oc-O2Oc-C-W-K*-F-O2Oc
7	Peptide 7: biotin-O2Oc-O2Oc-C-G-K-D-O2Oc-O2Oc
8	Glucose-modified HSA
9	Unmodified HSA
10	Glucose-modified BSA
11	Glyceraldehyde-modified BSA
12	Glyoxal-modified BSA
13	CML-BSA (reductive amination)
14	CEL-BSA (reductive amination)
15	Unmodified BSA
16	Glucose-modified IgG
17	Glyceraldehyde-modified IgG
18	Methylglyoxal-modified IgG
19	Glyoxal-modified IgG
20	CML-IgG (reductive amination)
21	CEL-IgG (reductive amination)
22	Unmodified IgG
23	Glucose-modified fibrinogen
24	Glucose-modified collagen
25	Unmodified collagen

The same targets were later used for evaluation of a chosen collection of expressed scFv:s.

K* = CML, O2Oc = 3,6-dioxaoctanoic acid.

### Screening of scFv:s expressed from selected phage display tracks

The screening procedure was performed as described by Persson *et al*. [29]. After three selection cycles, the scFv constructs from the resulting phage pools were subcloned into the expression vector pJB33 (kindly provided by Prof. A. Plückthun, University of Zürich, Switzerland) [32] using SfiI enzyme (New England Biolabs). The resulting constructs were transformed into *E*. *coli* XL1-Blue competent cells and grown overnight on LB agar-plates with 25 μg/mL chloramphenicol and 1% glucose at 37°C. Single colonies were picked and grown in LB-medium containing 25 μg/mL chloramphenicol and 1% glucose, overnight at 37°C, in 96-well plates. The overnight cultures were inoculated into new LB-medium containing 25 μg/mL chloramphenicol, and grown for 3.5 hours before induction with IPTG (final concentration 0.5 mM) and cultivation overnight at 37°C. Bacteria were separated from the scFv-containing solutions with centrifugation.

After separation from the bacteria, scFv-containing supernatants were printed three times spot-on-spot as earlier described [29], on top of six glycated and four non-glycated targets that were immobilized on an NHS-activated surface (Table 3). Proteins that were present in the mouse immunizations were included as positive screening targets, together with glucose-modified HSA and Fmoc-CML peptides that were included in the selection tracks. Negative targets were the corresponding unmodified structures. Binding was detected with 4 μg/mL monoclonal mouse anti-6xHis antibody (R&D Systems, Minneapolis, USA, cat. no. MAB050) and polyclonal Cy^™^5 AffiniPure Goat Anti-Mouse IgG H + L (Jackson ImmunoResearch Laboratories, PA, USA, cat. no. 115-175-146).

**Table 3:** Microarray-immobilized targets for spot-on-spot screening of scFv:s.

**Positive targets**
CML-BSA (reductive amination)
CEL-BSA (reductive amination)
CML-IgG (reductive amination)
CEL-IgG (reductive amination)
Glucose-modified HSA
Peptides 1–6
**Negative targets**
Unmodified BSA
Unmodified IgG
Unmodified HSA
Peptide 7

See Table 2 for peptide sequences.

*E*. *coli* XL1-Blue, expressing 20 clones that were chosen from the screening, were again expressed with IPTG. Eighteen of the 20 clones were successfully cultured. The resulting scFv-containing supernatants were then incubated on the same microarray-printed 25 glycated, and non-glycated, targets that were used in the phage selection array (Table 2) and detected with 4 μg/mL monoclonal mouse anti-6xHis antibody and polyclonal Cy^™^5 AffiniPure Goat Anti-Mouse IgG H+L.

### Sequencing

Selected clones were sequenced (GATC Biotech AG, Konstanz, Germany) and their sequences aligned.

### Further microarray evaluation of sequenced scFv:s

Bacterial supernatants from the sequenced clones, 1:500 mouse monoclonal anti-CML, KH011 raised against CML-KLH (Cosmobio, Tokyo, Japan) and 10 μg/mL recombinant sRAGE (Origene, Rockville, MD, USA), were further evaluated for binding to microarray-printed PepLib1 and PepLib2 on an NHS-activated surface (see Microarray printing of peptides and proteins). Binding scFv:s and 6xHis-tagged sRAGE were detected with 4 μg/mL monoclonal mouse anti-6xHis antibody and polyclonal Cy^™^5 AffiniPure Goat Anti-Mouse IgG (H+L). KH011 was detected with polyclonal Cy^™^5 AffiniPure Goat Anti-Mouse IgG (H+L).

### Dotblot

Standard dotblot on nitrocellulose was made with bacterial supernatant from the D1-B2 expression, KH011, mouse monoclonal anti-CEL KH025 raised against CEL-BSA (Cosmobio, Tokyo, Japan) and recombinant sRAGE. In short, dots with 50 mg/mL BSA incubated for one, two, three and four weeks with 0.5 M D-glucose or D-(-)-ribose in 37°C were dotted on a nitrocellulose membrane. Membranes were blocked with 5% milk in PBS. D1-B2 supernatant was diluted 1:2 and KH011/KH025 to 0.6 μg/mL with 0.05% Tween-20 in PBS. The prepared dilutions were incubated with the dotted membranes, on shake, for two hours. PBS, 0.05% Tween-20 was used as a negative control. After incubation, the dotblots were washed two times with 0.05% Tween-20 in PBS. D1-B2 binding was detected with mouse monoclonal anti-6xHis-HRP (R&D Systems, MN, Canada, cat. no. MAB050H) and KH011/KH025 binding was detected with goat polyclonal anti-mouse-IgG-HRP (Sigma Aldrich, Copenhagen, Denmark, cat. no. A4416) and luminol substrate on an Alfa Innotech, Fluor Chem FC2, Multi Image II.

### NiNTA purification

NiNTA purification of D1-B2, expressed in 100 mL scale, was performed according to standard procedures. Fractions eluted with 250 mM imidazol were controlled for purity with SDS-PAGE on Mini-PROTEAN^®^TGX^™^ 4–15% Precast gels and concentrations were determined using Micro BCA^™^ Protein Assay Kit.

### Microarray epitope-mapping of NiNTA-purified D1-B2 against PepLib3 and ProtLib1

Purified D1-B2, 30 μg/mL, was further analyzed against PepLib3 and ProtLib1, both immobilized onto an NHS-activated surface (see Microarray printing of peptides and proteins). For comparison, the same targets were incubated with 1:500 KH011 and 10 μg/mL recombinant 6×His-tagged sRAGE. D1-B2 and 6xHis-tagged sRAGE binding were detected with 4 μg/mL monoclonal mouse anti-6xHis antibody and polyclonal Cy^™^5 AffiniPure Goat Anti-Mouse IgG (H+L). KH011 was detected with polyclonal Cy^™^5 AffiniPure Goat Anti-Mouse IgG (H+L).

### Immunohistochemistry

Atherosclerotic plaques from ApoE-/- mice on C57BL/6 background (Taconic) that had been fed a cholesterol diet (0.15% cholesterol, 21% fat) from five weeks of age [36] were used in IHC with D1-B2. This study was carried out in strict accordance with the recommendations in the Guide for the Care and Use of Laboratory Animals of the National Institutes of Health. The Local Animal Care and Use Committee at Lund University approved the experimental protocol used in the study (Permit number: M200–01). All surgery was performed under anesthesia, and all efforts were made to minimize suffering. Twenty weeks old females were sacrificed by an intraperitoneal overdose of Hypnorm/Dormicum, and exsanguinated by cardiac puncture. Next, the mice were whole body perfused with phosphate buffer saline (PBS). Hearts were dissected and placed in Histochoice and were then embedded in OCT and sectioned into 10 μm sections using a cryostate according to previously published protocol [37]. Sections with subvalvular plaques were fixed in ice-cold acetone, permeabilized in 0.5% Triton X-100, and endogenous peroxidase was quenched with 1% H_2_O_2_ in PBS. Sections were then blocked with 10% mouse serum before addition of 10 μg/mL D1-B2 scFv, followed by 0.5 μg/mL biotin-labelled monoclonal mouse anti-6x-His antibody (Thermo Fisher Scientific, Massachusetts, USA, cat. no. MA121315BTIN). Antibody blocking was performed with 1:300 CML-BSA or with 1:35 CML-BSA-peptide 14, both in molar ratios and unmodified BSA or unmodified peptide 14 in the same concentrations. Detection was done with VECTASTAIN ABC HRP Kit and colour development was conducted by adding ImmPACT DAB Peroxidase (HRP) Substrate, counterstaining with Mayer’s hematoxylin.

A tissue microarray (TMA) from 82 pancreatic cancer patients was prepared as earlier described [38], using a TMArrayer semi-automated arraying device (Pathology Devices, Westminister, MD, USA). The study had been approved by the Ethics Committee of Lund University (ref nr 445/07). For immunohistochemical analysis, 4 μm TMA-sections were automatically pre-treated using the PT Link system and then stained in an Autostainer Plus (DAKO, Glostrup, Denmark) with 10 μg/mL D1-B2.

### Statistical analysis of microarray data

Microarray data were analyzed using Microsoft Office Excel macro files. Average RFU from the three spot triplets of each printed antigen were calculated together with in-spot standard deviations. In case of determination of positive or negative binding results, cutoffs for positive binding were defined according to cutoff = 1 × average RFU + (2 × in-spot stdev).

## Results

For the generation of scFv:s we used our previously published phage display based strategy and high-throughput microarray screening using protein and peptide libraries [29], where spleens from mice immunized with glycated proteins were used for construction of a phage display antibody library.

### Synthesis of target libraries

Four different libraries of peptide and protein targets were synthesized and used in selection and characterization of the antibodies (Table 4). Peptide libraries were made in a μmol scale using automated Fmoc solid-phase peptide synthesis (SPPS) with glycated lysine building blocks [31]. PepLib1 and the negative control PepLib2, covering the whole BSA sequence (Uniprot entry P02769) with CML-modified lysines, were synthesized as 20mers with a 10 amino acid overlap. A peptide library consisting of variants of peptide 14, ^131^DDSPDLPK*LK*PDPNTLCDEF^150^, from PepLib1 was synthesized in the same manner, constructing PepLib3. Full-length proteins were incubated with carbohydrates under various conditions, resulting in ProtLib1. Glycation was confirmed by observing browning of the reaction solutions. CML-modifications of glyoxal-modified HSA, glyoxal-modified fibrinogen, glyoxal-modified BSA, ribose-modified BSA and glucose-modified BSA, pH 10, were detected with KH011. Glycation of a chosen collection of BSA modifications was compared with sodium dodecyl sulfate polyacrylamide gel electrophoresis (SDS-PAGE) (S1 Fig). Ribose was the most effective glycating agent compared to glucose and fructose. Raising of pH also increased glycation. Detailed lists of library targets can be found in Supplemental data (S1–S4 Tables).

**Table 4:** A summary of the produced peptide and protein libraries.

Name	Content	Nr of targets	Sequence list
PepLib1	20mer peptides covering the full BSA sequence with carboxymethyl-modifications on each lysine residue	60	S1 Table
PepLib2	20mer peptides covering the whole BSA sequence	60	S2 Table
PepLib3	Peptides made from alterations of peptide 14 from PepLib1	65	S3 Table
ProtLib1	Carbohydrate-modified full-length proteins	74	S4 Table

### Selection and screening of antibodies

Selection of phage antibodies was divided into three parallel tracks, each focussed on different targets (Table 1). Parallel microarray analysis of the resulting phage pools from each selection cycle enabled concurrent observation of the binders generated. After two selection cycles, all phage pools contained binders from AGE-modified and unmodified IgG. Except these targets, track 3 also contained binders to peptide 5. After three selection cycles, all phage pools still contained binders from AGE-modified and unmodified IgG. Additionally, track 1 contained binders to CML-BSA, track 2 contained binders to CML- and carboxyethyl lysine (CEL)-BSA and track 3 contained binders to peptide 1 and 6 (Table 2). After conversion to single-clone 6xHis-tagged scFv:s, a total of 512 clones were picked, expressed and screened on a spot-on-spot microarray against six glycated and four non-glycated targets (Table 3) [39,40]. Analysis of the microarray data resulted in 20 clones that had a preference for the glycated over the non-glycated targets and were therefore chosen for further screening.

### Further screening against 25 targets

Chosen clones from the spot-on-spot scFv screening were expressed in 10 mL scale for in-solution incubation on targets immobilized on microarrays. Eighteen of the 20 clones successfully expressed scFv:s. Thirteen of the 18 clones had a preference for binding glycated targets when incubated on a microarray against 25 glycated, and non-glycated targets that were used in the phage selection array (Table 2). These clones were sequenced to verify monoclonality, resulting in nine unique scFv clones. These nine unique clones were cultured and expressed in 100 mL scale and were again tested against the 25 glycated targets. Four of these nine clones; D1-B2 and D2-D9 (originating from selection track 1) and E2-G6 and E2-A2 (originating from selection track 2), exhibited the most specific binding patterns to CML- and/or CEL- glycated targets, without binding to the corresponding unglycated targets (Table 5).

**Table 5:** Microarray binding patterns of nine chosen and sequenced scFv clones (supernatants from bacterial expression) against 25 glycated epitopes. Target nr# 1. Peptide 1, 2. Peptide 2, 3. Peptide 3, 4. Peptide 4, 5. Peptide 5, 6. Peptide 6, 7. Peptide 7, 8. Glucose-modified HSA, 9. Unmodified HSA, 10. Glyceraldehyde-modified BSA, 11. Glucose-modified BSA, 12. Glyoxal-modified BSA, 13. CML-BSA (reductive amination), 14. CEL-BSA (reductive amination), 15. Unmodified BSA, 16. Glucose-modified IgG, 17. Glyceraldehyde-modified IgG, 18. Methylglyoxal-modified IgG, 19. Glyoxal-modified IgG, 20. CML-IgG (reductive amination), 21. CEL-IgG (reductive amination), 22. Unmodified IgG, 23. Glucose-modified fibrinogen, 24. Glucose-modified collagen, 25. Unmodified collagen.

Target nr#	D1-B2	E2-A9	E2-G6	E2-A2	F2-G8	F1-E10	E2-E5	D1-D2	D2-D9
**1**.									
**2**.									
**3**.									
**4**.									
**5**.									
**6**.									
**7**.									
**8**.									
**9**.									
**10**.			X	X					X
**11**.									
**12**.	X		X	X		X			X
**13**.	X		X	X		X			X
**14**.	X		X	X					X
**15**.									
**16**.		X		X	X		X	X	
**17**.									
**18**.									
**19**.		X					X	X	
**20**.		X	X	X	X	X	X	X	
**21**.				X		X	X	X	
**22**.		X			X	X	X	X	
**23**.									
**24**.									
**25**.									

Cutoff for positive binding was defined as an RFU value of (1×average RFU + 2×in-spot stdev) from the same incubation. For peptide sequences, see Table 2.

### Evaluation of binding against CML-BSA peptide library PepLib1

Supernatants from bacterial expression of the four selected clones (D1-B2, D2-D9, E2-G6 and E2-A2) were evaluated in a microarray format against the epitopes of the CML-BSA peptide library (PepLib1) and the corresponding non-AGE modified library (PepLib2) (S2 Fig). D1-B2 bound to the highest number of glycated peptides from PepLib1, but not to the peptides from the corresponding negative library. The scFv clones D2-D9, E2-G6 and E2-A2 only bound to one or two of the PepLib1 peptides each. D2-D9 showed high binding to one of the unglycated BSA peptides and additionally it showed binding to the unglycated full-length BSA control. E2-G6 and E2-A2 showed no binding to the negative BSA peptide library. Thus, D1-B2 was chosen for purification and further characterization of binding epitopes because of its ability to detect several of the glycated epitopes and its low binding to the unglycated peptides.

### Comparison between D1-B2, commercial monoclonal anti-CML and recombinant sRAGE

**Fig 1:**
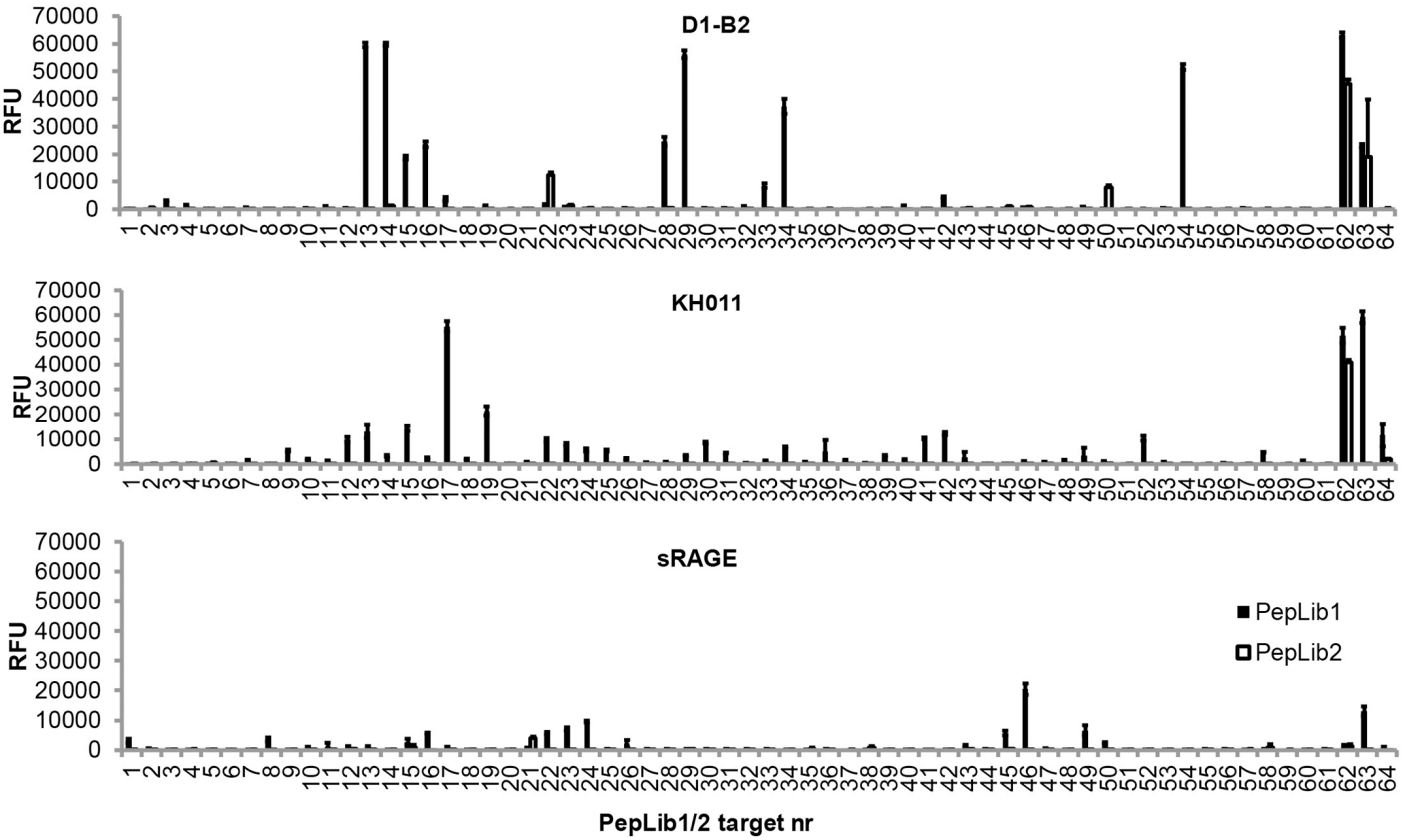
Binding of D1-B2, KH011, and recombinant sRAGE to PepLib1 and PepLib2 printed on microarrays. Histograms are plotted as relative fluorescense units (RFU) against peptide number. For specifications of peptides, see S1 Table for PepLib1 and S2 Table for PepLib2. In-spot standard deviations are displayed in the bars. For quantified data of the binding of D1-B2 to these libraries, see S5 and S6 Tables, respectively.

NiNTA-purified D1-B2, recombinant soluble RAGE (sRAGE) and commercial monoclonal mouse anti-CML antibody (KH011) were evaluated against PepLib1 and 2 (Fig 1). D1-B2 was added in a high concentration (30 μg/mL) to reveal any possible background binding. Maximal binding signals showed over-saturation of peptides 13, 14, 29, 54 from PepLib1. Also peptides 15, 16, 28, and 34 from the same library generated high signals. Low binding could be seen to peptides 22 and 50 from PepLib2. KH011 and sRAGE gave distinctively different binding patterns to PepLib1 than D1-B2 and did not bind to PepLib2. Incubation of sRAGE resulted in very low binding signals.

### The binding properties of D1-B2 were characterized with glycated full-length protein library ProtLib1 printed on a microarray

**Fig 2:**
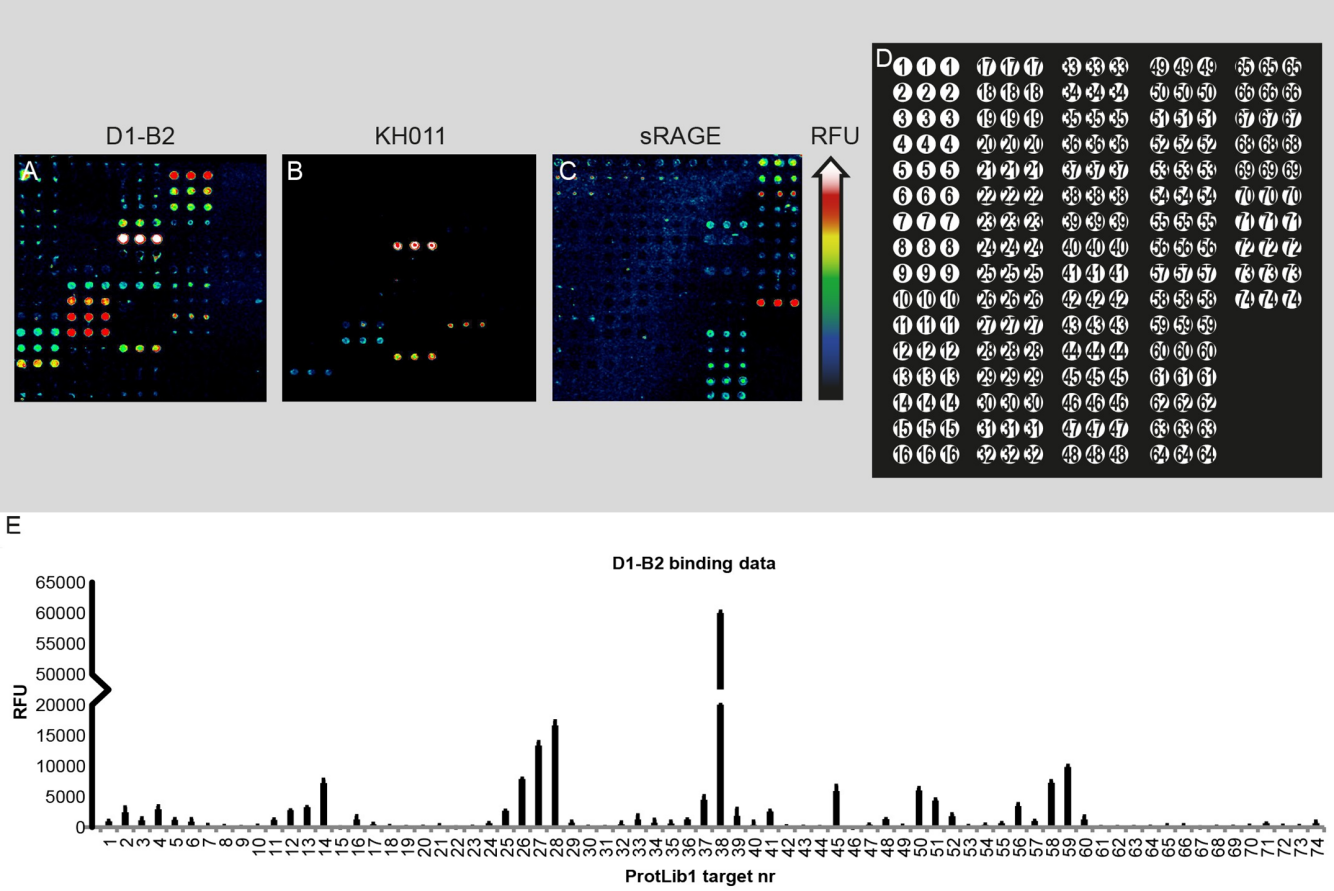
Microarray binding of D1-B2, KH011, and recombinant sRAGE to ProtLib1. (A) D1-B2, (B) KH011, (C) recombinant sRAGE, (D) Printing layout, and (E) quantified data of the binding signal from D1-B2. For descriptions of the modified proteins in ProtLib1, see S4 Table. For quantified data on the binding of D1-B2 to ProtLib1, see S8 Table. Order of the colors indicating relative fluorescence units can be seen beside the array pictures. In-spot standard deviations are displayed in the bars.

The 74 targets of the glycated full-length protein library ProtLib1 (S4 Table) were printed on a microarray and incubated with D1-B2 (Fig 2A). KH011 and sRAGE were incubated on the same library for comparison (Fig 2B and 2C). Glyoxal, which is expected to give CML modifications, gave the highest signals from D1-B2 (target nr. 38, 45, and 59, Fig 2A and 2E, S8 Table). Of the different sugars that had been used as glycating agents (fructose, glucose, and ribose), D1-B2 showed strongest binding to ribose modifications (target nr. 28, Fig 2A and 2E, S8 Table), which were also identified to be the most highly glycated proteins in SDS-PAGE of modified BSA (S1 Fig). Glycations generated at higher pH also gave a stronger signal with D1-B2 compared to neutral pH (target nr. 14 and 7 respectively, Fig 2A and 2E, S8 Table). Higher signals were obtained from samples with longer incubation times compared to shorter incubation times. KH011 (Fig 2B) and sRAGE (Fig 2C) showed binding to fewer glycated epitopes than D1-B2.

### Epitope mapping of D1-B2 with PepLib3

**Fig 3:**
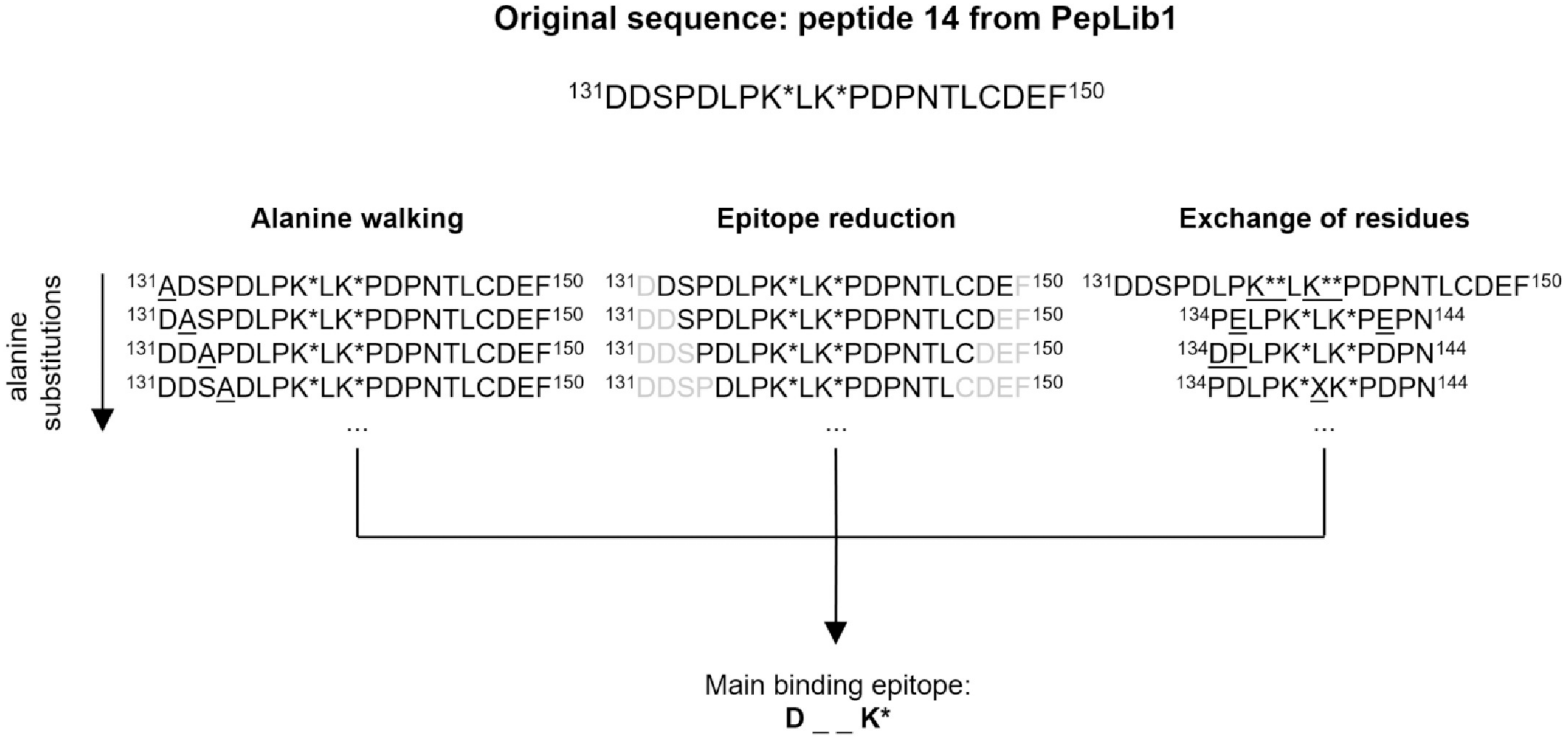
Epitope mapping strategy for characterization of D1-B2 binding with PepLib3. Epitope mapping of D1-B2 was performed with the PepLib3 peptide library, constructed from peptide 14 of PepLib1. Three epitope mapping strategies were performed; alanine walking, eptitope reduction and exchange of residues. The three strategies resulted in the identification of the main binding epitope of D1-B2: **D** _ _ K*. K* = CML, K** = succinylated lysine.

PepLib3 (S3 Table) was constructed from variations of the sequence of peptide 14 from PepLib1 (S1 Table), ^131^DDSPDLPK*LK*PDPNTLCDEF^150^, as this glycated peptide showed maximal binding signal from detection with D1-B2 (Fig 1, S5 Table); and at the same no binding was seen towards the same unglycated peptide from PepLib2 (Fig 1, S6 Table). Variations of a shorter version of peptide 14, ^134^PDLPK*LK*PDP^144^ (peptide 15/38 in PepLib3, S3 Table) were also included in the evaluation. The resulting binding data showed the contribution of each individual amino acid in the CML-BSA peptide 14 sequence and revealed the main binding site of D1-B2 (Fig 3, S7 Table).

Epitope mapping was performed using three strategies; alanine walking, epitope reduction and exchange of residues (Fig 3).

In the alanine walking, every amino acid in the original peptide sequence was exchanged for an alanine residue to see how that specific amino acid affected D1-B2 binding. Exchange of either the CML residue at position 138 or the aspartic acid residue on position 135 for alanine almost removed the binding signal (peptide 6 and 9, S7 Table). Exchange of the threonine on position 145, the leucine on position 146 and the aspartic acid on position 148 to an alanine, all reduced the signal to some extent in increasing order (peptide 51, 52, and 54, S7 Table). The remaning amino acids did not seem to affect binding.

Reduction of the backbone around the CML residue so that the aspartic acid on position 135 and the threonine on position 145 were excluded almost completely prevented binding by D1-B2 (peptide 60, S7 Table).

Moving of the aspartic acid at position 135 to a another position almost completely deleted binding (S7 Table). Moving of the other aspartic acid at position 142 did not reduce binding to the same extent (S7 Table). At neutral pH, CML modification exchanges a positive charge on an unmodified lysine to a negative charge. Exchange of the CML residues for succinylated lysines, that also alternates the positive charge on unmodified lysines to negative charges at neutral pH, eliminated binding signal (peptide 65, S7 Table). Exchange of aspartic acid for glutamic acid, which also has a negative charge at neutral pH, strongly reduced the signal (peptide 28, S7 Table). Thus, it can be confirmed that the binding of D1-B2 is not only dependent on negative charges, but is specific to CML.

Alternation of the amino acid between the two CML residues of CML-BSA peptide 14 to all 20 amino acids gave varying results, e.g. aromatic amino acids gave a higher binding signal, whereas arginine, methionine and aspartic acid reduced the signal (peptide 29–48, S7 Table). KH011 did not bind to the PepLib3 epitope mapping library (S3 Fig).

**Fig 4:**
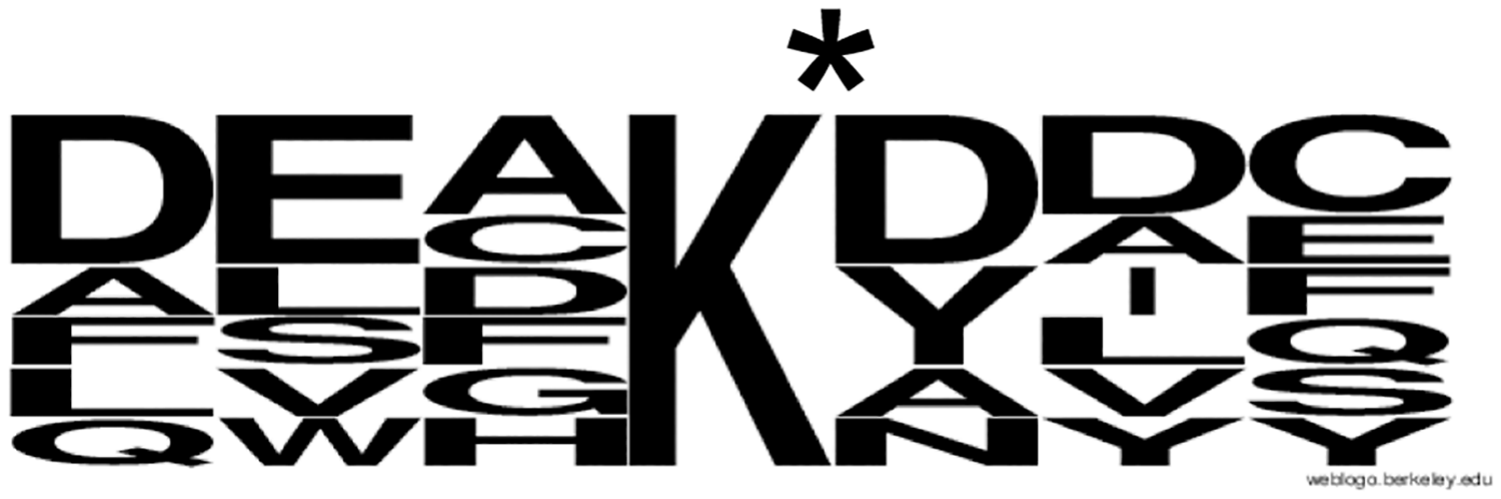
Sequence logo of the CML-sites of the peptides in PepLib1, which D1-B2 bound to. The importance of a D residue, three positions N-terminally from CML is confirmed in this figure.

In total, epitope mapping showed that D1-B2 has a preference for the epitope D_ _K*, where K* = CML (Fig 3). Creating a sequence logo from the CML epitopes of the CML-BSA peptides from PepLib1 that D1-B2 bound to further stated this preferred sequence (Fig 4).

### Dot blot

Further characterization of D1-B2 was performed with a nitrocellulose dot blot against BSA, glycated with glucose or ribose at 37°C for one, two, three, and four weeks (S4 Fig). D1-B2 showed a high binding signal against ribose-modified BSA, compared to KH011 and anti-CEL (KH025) monoclonal mouse antibodies. Binding signal increased with longer BSA glycation times. Glucose-modified BSA did not give any antibody binding in this assay.

### Specific binding of D1-B2 to ApoE-/- mouse atherosclerotic plaques

**Fig 5:**
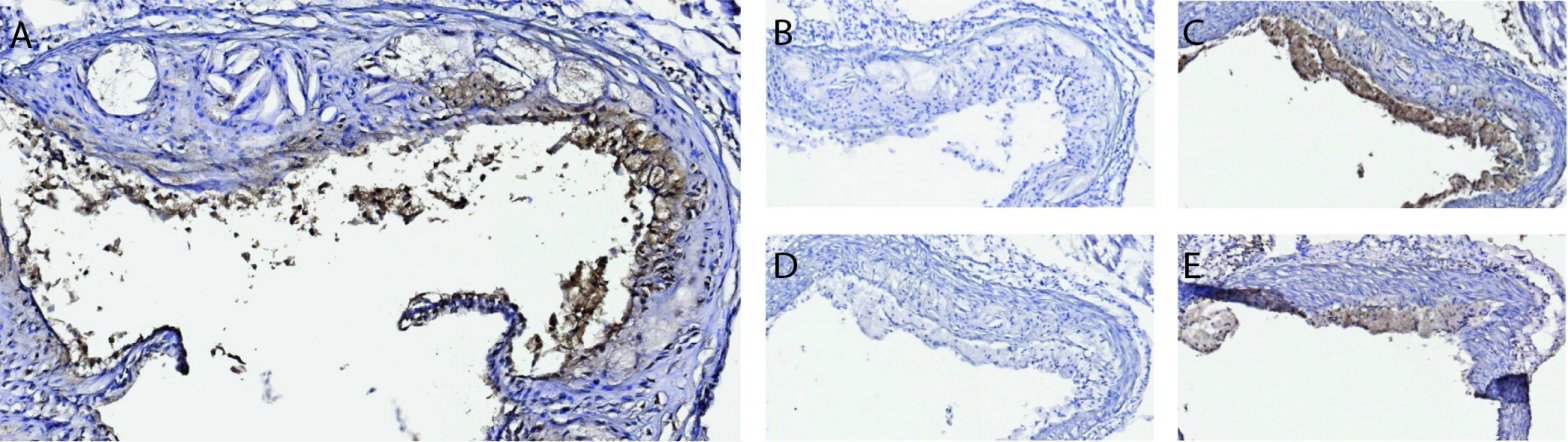
Immunohistochemistry with D1-B2 binding to atherosclerotic aorta plaques from ApoE-/- mice. (A) Unblocked D1-B2, (B) Blocking with 1:300 CML-BSA, (C) Blocking with 1:300 unmodified BSA, (D) Blocking with 1:35 CML-peptide nr. 14 from PepLib1, (E) Blocking with 1:35 unmodified peptide nr. 14 from PepLib2. All pictures were taken at 15 x magnification. Specific binding to plaques and not to the surrounding aorta wall was observed. Staining was removed by blocking with CML-modified full-length BSA and CML-peptide, but not with the same unmodified targets.

Immunohistochemistry was performed on sections from atherosclerotic plaques, prepared from ApoE-/- mice fed with a high-fat diet, a model that would be expected to have a high risk of AGE modifications. D1-B2 bound selectively to plaques and not to the surrounding aorta wall or the muscle tissue (Fig 5A). Blocking with CML-BSA and CML-BSA-peptide 14 removed specific staining (Fig 5B and 5D). Blocking with unmodified BSA (Fig 5C) and unmodified BSA peptide 14 (Fig 5E) did not reduce staining.

### D1-B2 bound to distinctive structures on a tissue microarray from pancreatic cancer patients

**Fig 6:**
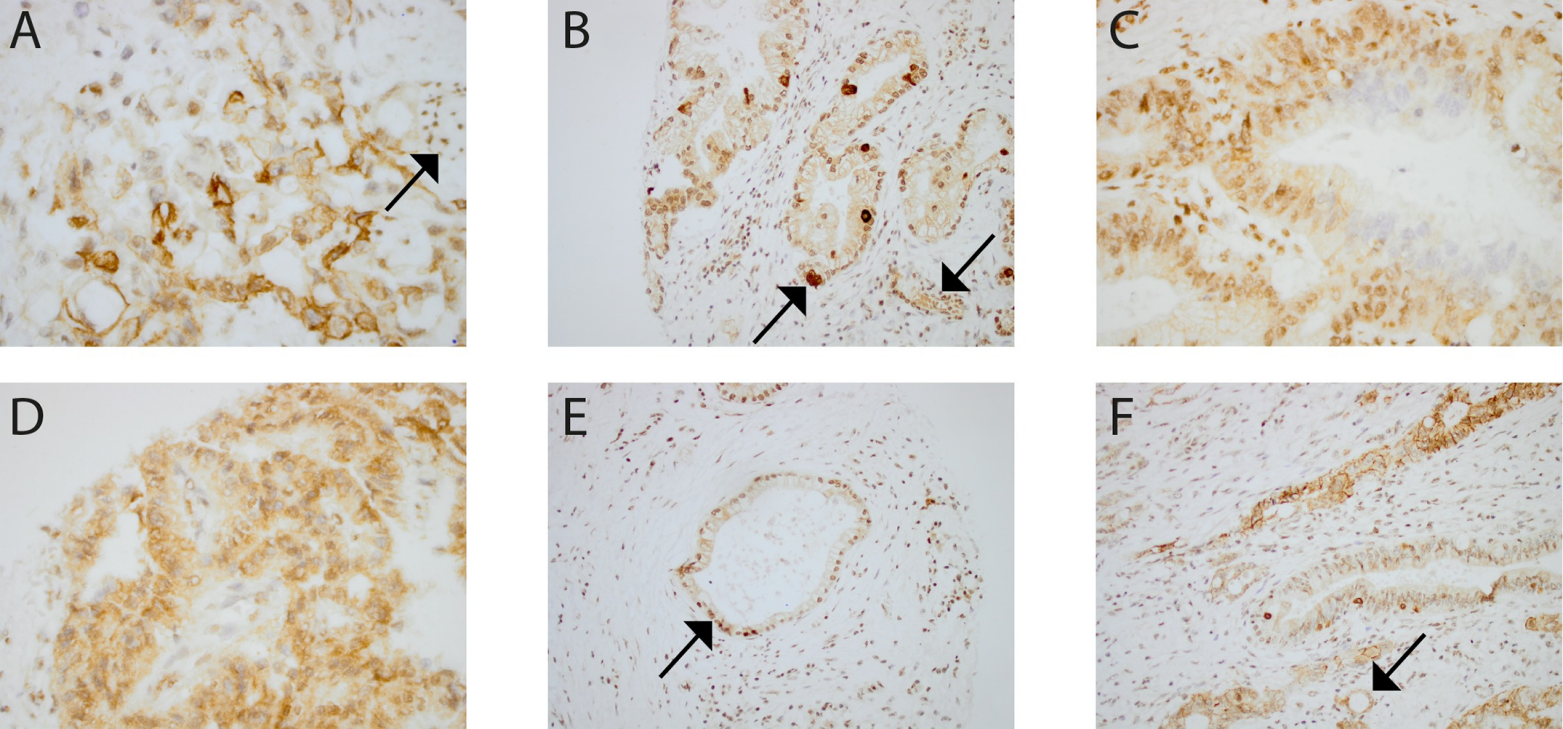
Examples of IHC staining from D1-B2 on a human pancreatic cancer TMA. (A) Diverse staining of nucleus, cytoplasm, and cell membranes. Arrow: Staining of a collection of immune cells can also be observed to the right in the picture. 40 x magnification. (B) Staining of cells surrounding a canal, formed in the tissue. Both cytoplasm and nucleus are colored. Arrow: Distinct dark spots, that could be interpreted as heavily glycated cells, appear among the other colored cells. Arrow: Collections of colored immune cells are present. The rest of the cell matrix is not colored to the same extent. 10 x magnification. (C) The outside of a cell formation has been stained in both cytoplasm and nucleus. The inside of the formation remains uncolored. 20 x magnification. (D) Staining of cytoplasm but, in most cells, not of nucleus. 10 x magnification. (E) Arrow: Staining of both cytoplasm and nucleus of cells surrounding what seems to be a necrotic area. The rest of the cell matrix is not colored to the same extent. 10 x magnification. (F) Contains all the patterns observed in Fig 5A–5E. Arrow: Formation of cavities in the areas especially rich in glycations are illustrated. These patterns can also be seen to some extent in Fig 5A. 20 x magnification.

A TMA was prepared from 82 tumor tissues from patients diagnosed with pancreatic cancer. The tumor tissue sections, each from one individual patient, were compared with IHC staining by D1-B2. All tissues stained positively, but there was a large variation observed in both staining patterns and staining intensity (Fig 6A–6F). In a number of pancreatic cancer cells, both the nuclei and cytoplasms were stained. In other cells the cytoplasms, but not the nuclei were stained. An increase in staining intensity could be seen around arteries and on surfaces lining the pancreatic ducts. Distinct dark spots and formation of cavities was also observed in some of the stained tissues. In a number of tissues, clusters of stained immune cells were present.

## Discussion

In this study, a phage display platform was used for selection of scFv:s binding to specific glycated epitopes. Extensive screening and evaluation against a large collection of protein and peptide antigens was performed using a microarray approach in parallel with the antibody development process [29]. This approach enabled early high-throughput detection of specific AGE-binders from a large pool of clones, unlike hybridoma technology.

In vitro glycated proteins were used both in the immunizations and in the microarray evaluations. BSA has been a model protein in many studies on AGE and was therefore chosen for in vitro glycation. The most prevalent blood proteins were also included in the glycation reactions because of their high exposure to carbohydrates in vivo. Collagen was included since it is longlived and therefore has a risk of accumulating more glycated residues.

Mouse immunizations for construction of a phage library were performed with the aim to obtain a broad and immature antibody response. Therefore, a diverse group of AGE-modified proteins were included in the immunizations and only one immunization boost was applied. Selections were then performed in three tracks focusing on CML- or CEL-modified proteins that were included in the immunizations and on Fmoc-CML peptides aiming to select binders to a small and defined epitope.

Parallel microarray analysis of the phage stocks resulting from the selection cycles enabled detection of the obtained binders to unmodified and AGE-targets. After both selection cycle 2 and 3, all phage pools contained binders to both modified and unmodified human IgG. Since AGE-modifed human IgG was included in the mouse immunizations, an immune response against this protein was expected. Pre- or counter-selection with human IgG could have been used to remove IgG binders. Since the selection rounds also generated binders to AGE-modifications, unspecific IgG binders were expected to be discarded during screening.

Screening was performed against six modified and four unmodified targets included in the selection tracks, to be able to identify which clones that possessed the desired binding characteristics. Twenty clones were chosen after the screening process basded on their binding to CML- and CEL-modified targets. Seven of the 20 clones that were picked out from the screening array were discarded after showing to low expression or after detecting undesired binding properties when re-analyzed in solution. Not all clones of the clones included in the spot-on-spot screening will have the desired qualities for large-scale culturing. This step therefore becomes a test of which clones that are suitable for further evaluation. The clones that were able to be expressed in solution were incubated against 25 targets on array. A number of clones that in screening appeared to be specific to CML modification, now bound to unmodified IgG and were classified as false positives. The spot-on-spot screening and the re-test through incubation with scFv:s in solution are two different assay setups, which explains the difference in results. In addition, spot-on-spot screening was performed with bacterial supernatants from expression in 96-well-plates, while the incubation against 25 targets was performed with supernatants expressed in larger scale. A significant difference in antibody concentrations could therefore not be excluded, which could give differences in binding results.

A number of clones that originated from selection track 2, which was directed to CEL-modifications, also bound to CML-modified proteins when evaluated against the 25 selection targets. Since CEL only is differentiated from CML by an additional methylene group, it is not unlikely to obtain cross-reactive clones. Pre- or counter-selection with CEL-modified proteins in track 1 and with CML-modified proteins in track 2 would perhaps have prevented the selection of cross-reactive clones. There is a risk though that this strategy would also remove most of the phage pool.

BSA was used as a scaffold for PepLib1 which was constructed for mapping of epitopes of CML-binding monoclonal antibodies. BSA was chosen because of its presence in the immunization solution and since several of the chosen clones bound to CML-BSA on array. A CML-BSA peptide library was therefore expected to contain the binding epitopes of many of the selected binders.

After selection, screening and evaluation, D1-B2 was chosen as the best candidate for further characterization, because of its binding to the largest number of glycated peptides and proteins, without high binding to the respective negative controls. The ability to bind a number of different targets not only enables a possibility of usage in many different applications, but also indicates a biological relevance of the binding epitope. Purified D1-B2 was re-tested against PepLib1 and 2 together with KH011 and recombinant sRAGE (Fig 1). D1-B2 was added in a high concentration (30 μg/mL) to reveal possible background binding. Signals were above the maximal detection limit for PepLib1 peptides 13, 14, 29 and 54, that all contained an aspartic acid three positions N-terminally from a CML residue. Low binding signals could be seen to PepLib2 peptides 22 and 50. Although weak binding to these two peptides, no binding could be seen to full-length unmodified BSA in any step of the evauation process (Table 5, Figs 1 and 2).

The binding patterns of the commercial anti-CML monoclonal antibody KH011 differed completely from the binding of D1-B2. Peptide 13, 14, 29 and 54 that resulted in maximal binding signals from D1-B2 incubation were not bound by KH011 (Fig 1). Not unexpectedly, KH011 did not bind to the epitope mapping library PepLib3 that was developed from peptide 14 (S3 Fig). These results further state that D1-B2 contributes with new detection possibilities of CML-sites and reveal the importance of knowing what epitope an antibody binds to. Purchasing a commercial monoclonal antibody against such a small carbohydrate modification as CML will not enable detection of all modified sites.

Incubation of sRAGE against the AGE libraries generated very low binding signals in general. This can be a matter of concentration, but it is also possible that the microarray format that the antigens are presented in is disadvantageous for the binding of this receptor. RAGE binds to negatively charged residues through a positively charged pocket [41]. Normalization of the binding signals presented a clearer image of the differences in binding patterns between D1-B2, KH011 and sRAGE (S5 Fig).

When immobilizing such large collections of targets on NHS surfaces, using microarray printing, there is a minor variation in printing quality between spots. This results in differences in binding signals from the evaluated antibodies. This issue is still overweighed by the large amount of information that is obtained in one experiment from microarray analysis.

D1-B2 was selected from a track that was directed against CML (Table 1, track 1). The binding data from incubation of the newly produced scFv on the microarray-printed epitope-mapping library PepLib3 showed that the core epitope was a CML with an aspartic acid residue three positions N-terminally (Fig 3). However, evaluation showed some binding to the very similar modification CEL, although to a much lower level than to CML (Table 5, Fig 2E). The cross-reactivity might be explained by the similarities between the structures. CEL only has one additional methylene group compared to CML. For diagnostics and detection of certain immunogenic epitopes though, this small difference is not necessarily a drawback. Both CML and CEL alter the positively charged unmodified lysine residue to a negatively charged residue and could therefore possibly make the same structural changes on the protein.

From the phage selection to the final D1-B2 epitope mapping, both peptides with Fmoc-CML building blocks and in vitro glycated proteins were used in the microarray evaluations. Printing of an antibody target on a microarray surface, results in a presentation that differs from biological tissues. By using both peptides and full-length proteins the possibility of selecting AGE-specific clones could be increased. Peptides containing the CML building block provide a dense presentation of glycations. That can be advantageous for selecting binders to such small carbohydrate antigens, which are often hard to develop strong binders against. Full-length proteins have structures that would be more similar to a biological presentation.

After characterizing D1-B2 using glycated antigens presented on an NHS surface, the new clone was further used in IHC on biological material. AGEs have been indicated to be present, and contribute to the formation of atherosclerotic plaques in a number of studies [42–44]. Positive staining of ApoE-/- mouse plaques both confirms the ability of D1-B2 to detect CML and the presence of the characterized CML epitope in this mouse model (Fig 5).

A much less studied phenomenon is the presence of AGE in cancer. D1-B2 stained diverse structures, including both the nucleus and/or cytoplasm in pancreatic cancer tissues (Fig 6). Distinct dark spots, which could be interpreted as heavily glycated cells, were observed in a few tissues. Collections of stained immune cells were present in several cases. Formation of cavities was detected in tissues that seemed to be heavily glycated. Similar AGE IHC staining patterns have been observed in other cancer tissue studies [45,46]. Increased glucose uptake and metabolism, which often are observed in tumors, may partially explain the presence of AGE in cancer. The role of AGE in cancer is today not fully understood, but the observed positive staining suggests a potential usage in IHC.

One possible next step in this project would be staining of a larger human cancer tissue collection with parallel staining of RAGE and glyoxalase. In this way both the presence of AGE structures, the biological defense against them and the potential of RAGE-activated inflammation could be compared. It would also be advantageous if the stained material included records of clinical data that could be linked to the staining results. Another possibility would be to label D1-B2 for in vivo imaging in animal models to see distribution after injection. If the new antibody proves to reach tissues rich in AGE structures, it could be used as a transporter of substances for therapies against AGE. This developed anti-CML antibody also provides a candidate scaffold for building new phage libraries for selection of new antibodies with specificities to alternative AGE epitopes.

In summary, D1-B2 is a scFv monoclonal antibody enabling detection of specific CML epitopes. To our knowledge, this is the first time the binding pattern of a monoclonal anti-AGE antibody has been so thoroughly characterized. This study is a promising illustration on how to generate new epitope-specific antibodies with potential use in diagnostic and therapeutic applications of AGE-related diseases.

## Supporting information

S1 Fig SDS-PAGE of glycated BSA samples, included in ProtLib1. As the glycation reaction proceeds, an increase in mass can be observed (here indicated by a shorter travelling distance on the gel). Samples: (1) BSA in 0.5 M glucose, 37°C, pH 7.2, start, (2) BSA in 0.5 M glucose, 37°C, pH 7.2, 1 week, (3) BSA in 0.5 M glucose, 37°C, pH 7.2, 2 weeks, (4) BSA in 0.5 M glucose, 37°C, pH 7.2, 3 weeks, (5) BSA in 0.5 M glucose, 37°C, pH 7.2, 4 weeks, (6) BSA in 0.5 M ribose, 37°C, pH 7.2, start, (7) BSA in 0.5 M ribose, 37°C, pH 7.2, 1 week, (8) BSA in 0.5 M ribose, 37°C, pH 7.2, 2 weeks, (9) BSA in 0.5 M ribose, 37°C, pH 7.2, 3 weeks, (10) BSA in 0.5 M ribose, 37°C, pH 7.2, 4 weeks, (11) BSA in 0.5 M fructose, 37°C, pH 7.2, start, (12) BSA in 0.5 M fructose, 37°C, pH 7.2, 1 week, (13) BSA in 0.5 M fructose, 37°C, pH 7.2, 2 weeks, (14) SeeBlue Prestained standard (Invitrogen), (15) BSA in 0.5 M fructose, 37°C, pH 7.2, 3 weeks, (16) BSA in 0.5 M fructose, 37°C, pH 7.2, 4 weeks, (17) BSA in 0.5 M glucose, 37°C, pH 10, start, (18) BSA in 0.5 M glucose, 37°C, pH 10, 1 week, (19) BSA in 0.5 M glucose, 37°C, pH 10, 2 weeks, (20) BSA in 0.5 M glucose, 37°C, pH 10, 3 weeks, (21) BSA in 0.5 M glucose, 37°C, pH 10, 4 weeks, (22) BSA in 0.5 M glucose, 50°C, pH 7.2, start, (23) BSA in 0.5 M glucose, 50°C, pH 7.2, 1 week, (24) BSA in 0.5 M glucose, 50°C, pH 7.2, 2 weeks, (25) BSA in 0.5 M glucose, 50°C, pH 7.2, 3 weeks, (26) BSA in 0.5 M glucose, 50°C, pH 7.2, 4 weeks.
(TIF)

S2 Fig Microarray binding patterns of four chosen scFv clones to PepLib1 and to PepLib2. (A) Clone D1-B2 against PepLib1, (B) Clone D2-D9 against PepLib1, (C) Clone E2-A2 against PepLib1, (D) Clone E2-G6 against PepLib1, (E) Clone D1-B2 against PepLib2, (F) Clone D2-D9 against PepLib2, (G) Clone E2-A2 against PepLib2, (H) Clone E2-G6 against PepLib2, (I) Microarray printing layout. Order of the colours indicating relative fluorescence units can be seen beside the array pictures.
(TIF)

S3 Fig Microarray binding of D1-B2 and KH011 to PepLib3. (A) D1-B2, (B) KH011, and (C) microarray printing layout. D1-B2 gives a significantly higher signal to a large part peptides of PepLib3 compared to KH011, indicating a completely different binding pattern. For peptide sequences included in PepLib3, see S3 Table. Order of the colours indicating relative fluorescence units can be seen beside the array pictures.
(TIF)

S4 Fig Dotblot of D1-B2, KH011 and KH025 against BSA glycated with glucose or ribose. (A) D1-B2, (B) negative control (no antibody), (C) KH011, (D) KH025, (E) antigen positions on dotblot.
(TIF)

S5 Fig D1-B2, KH011 and sRAGE binding to PepLib1 and PepLib2, illustrated with normalized signals. Normalized signals in % were calculated by dividing each RFU value with the maximal RFU value in the same analysis and then multiplying with 100.
(TIF)

S1 Table PepLib1 sequences. (PDF)

S2 Table PepLib2 sequences. (PDF)

S3 Table PepLib3 sequences. (PDF)

S4 Table ProtLib1 target specifications. (PDF)

S5 Table D1-B2 binding data to PepLib1. (PDF)

S6 Table D1-B2 binding data to PepLib2. (PDF)

S7 Table D1-B2 binding data to PepLib3. (PDF)

S8 Table D1-B2 binding data to ProtLib1. (PDF)
