# Supplementary figures and images for: A novel monoclonal antibody targeting carboxymethyllysine, an advanced glycation end product in atherosclerosis and pancreatic cancer
Source: PLoS One. 2018 Feb 8;13(2):e0191872. doi: 10.1371/journal.pone.0191872 (PMC5805250; doi:10.1371/journal.pone.0191872)

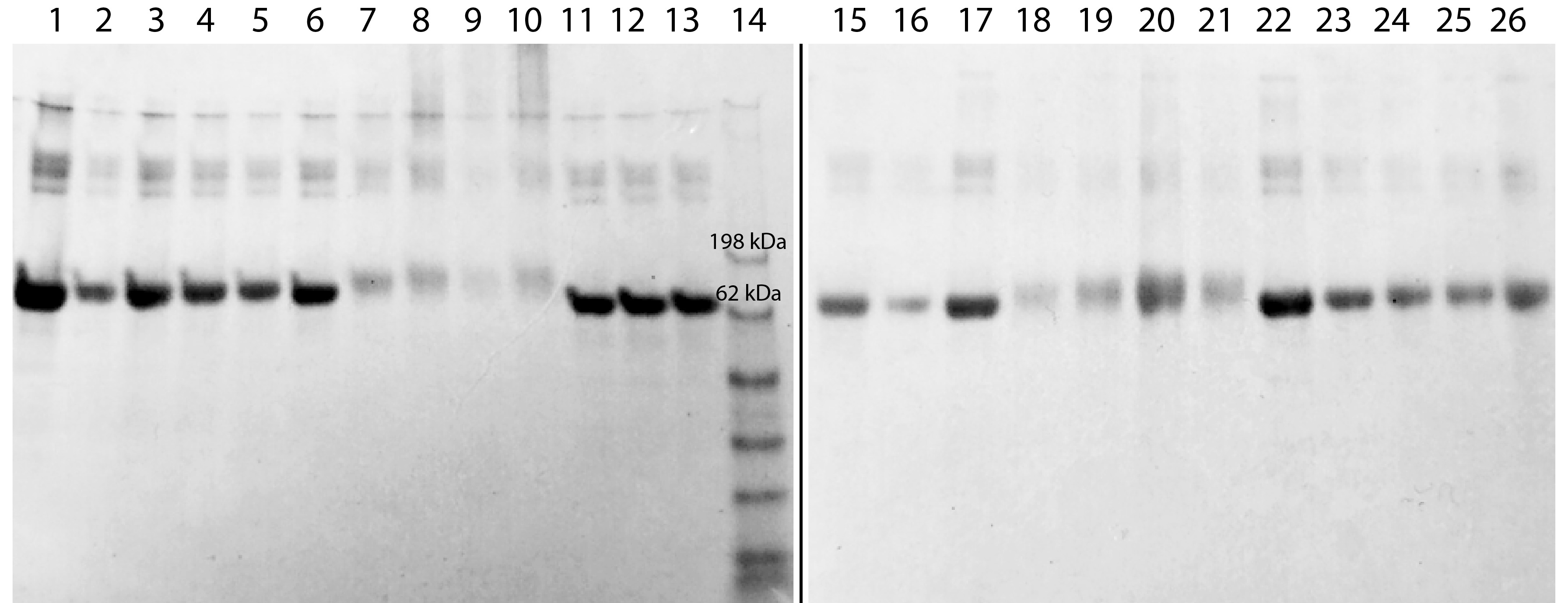

Supplement: S1 Fig — As the glycation reaction proceeds, an increase in mass can be observed (here indicated by a shorter travelling distance on the gel). Samples: (1) BSA in 0.5 M glucose, 37°C, pH 7.2, start, (2) BSA in 0.5 M glucose, 37°C, pH 7.2, 1 week, (3) BSA in 0.5 M glucose, 37°C, pH 7.2, 2 weeks, (4) BSA in 0.5 M glucose, 37°C, pH 7.2, 3 weeks, (5) BSA in 0.5 M glucose, 37°C, pH 7.2, 4 weeks, (6) BSA in 0.5 M ribose, 37°C, pH 7.2, start, (7) BSA in 0.5 M ribose, 37°C, pH 7.2, 1 week, (8) BSA in 0.5 M ribose, 37°C, pH 7.2, 2 weeks, (9) BSA in 0.5 M ribose, 37°C, pH 7.2, 3 weeks, (10) BSA in 0.5 M ribose, 37°C, pH 7.2, 4 weeks, (11) BSA in 0.5 M fructose, 37°C, pH 7.2, start, (12) BSA in 0.5 M fructose, 37°C, pH 7.2, 1 week, (13) BSA in 0.5 M fructose, 37°C, pH 7.2, 2 weeks, (14) SeeBlue Prestained standard (Invitrogen), (15) BSA in 0.5 M fructose, 37°C, pH 7.2, 3 weeks, (16) BSA in 0.5 M fructose, 37°C, pH 7.2, 4 weeks, (17) BSA in 0.5 M glucose, 37°C, pH 10, start, (18) BSA in 0.5 M glucose, 37°C, pH 10, 1 week, (19) BSA in 0.5 M glucose, 37°C, pH 10, 2 weeks, (20) BSA in 0.5 M glucose, 37°C, pH 10, 3 weeks, (21) BSA in 0.5 M glucose, 37°C, pH 10, 4 weeks, (22) BSA in 0.5 M glucose, 50°C, pH 7.2, start, (23) BSA in 0.5 M glucose, 50°C, pH 7.2, 1 week, (24) BSA in 0.5 M glucose, 50°C, pH 7.2, 2 weeks, (25) BSA in 0.5 M glucose, 50°C, pH 7.2, 3 weeks, (26) BSA in 0.5 M glucose, 50°C, pH 7.2, 4 weeks. (TIF) [file pone.0191872.s001.tif]

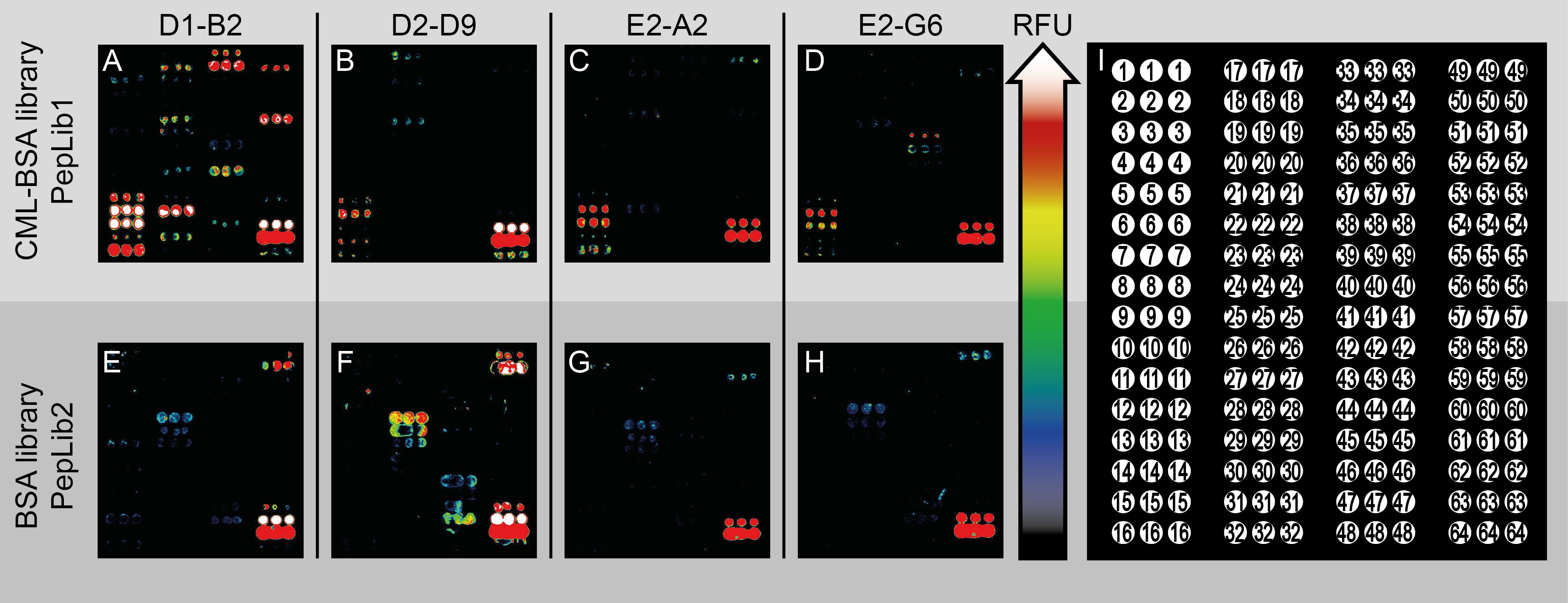

Supplement: S2 Fig — (A) Clone D1-B2 against PepLib1, (B) Clone D2-D9 against PepLib1, (C) Clone E2-A2 against PepLib1, (D) Clone E2-G6 against PepLib1, (E) Clone D1-B2 against PepLib2, (F) Clone D2-D9 against PepLib2, (G) Clone E2-A2 against PepLib2, (H) Clone E2-G6 against PepLib2, (I) Microarray printing layout. Order of the colours indicating relative fluorescence units can be seen beside the array pictures. (TIF) [file pone.0191872.s002.tif]

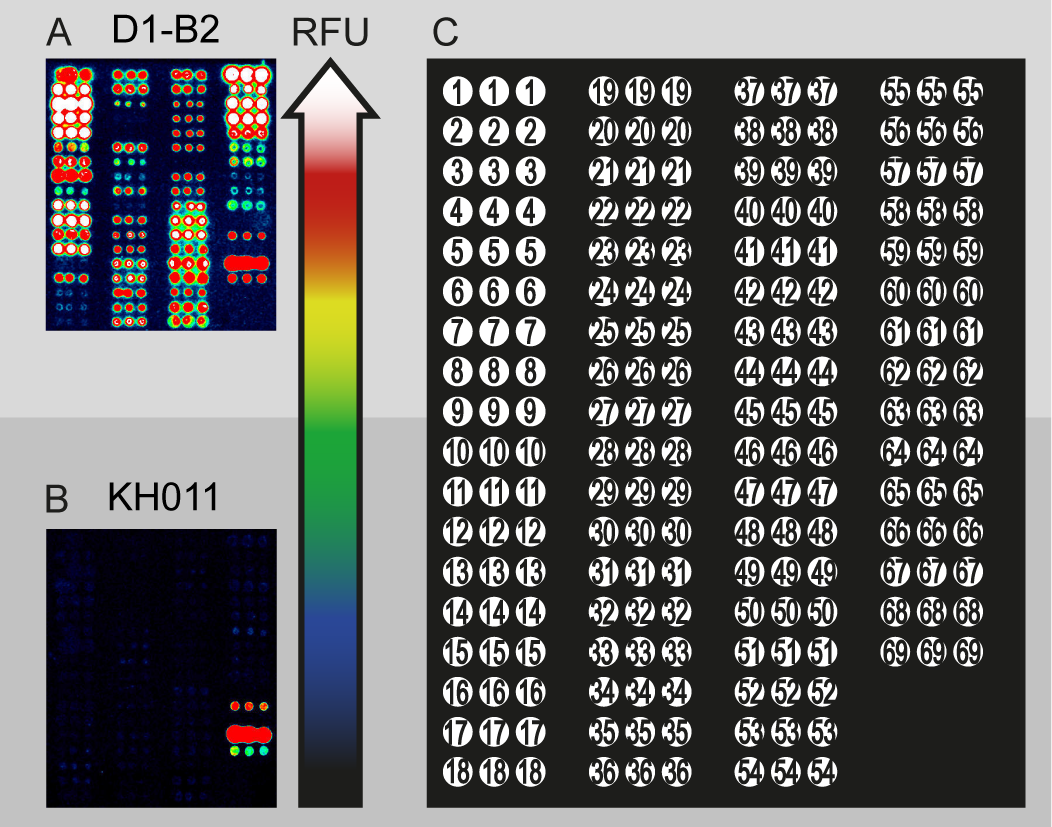

Supplement: S3 Fig — (A) D1-B2, (B) KH011, and (C) microarray printing layout. D1-B2 gives a significantly higher signal to a large part peptides of PepLib3 compared to KH011, indicating a completely different binding pattern. For peptide sequences included in PepLib3, see S3 Table. Order of the colours indicating relative fluorescence units can be seen beside the array pictures. (TIF) [file pone.0191872.s003.tif]

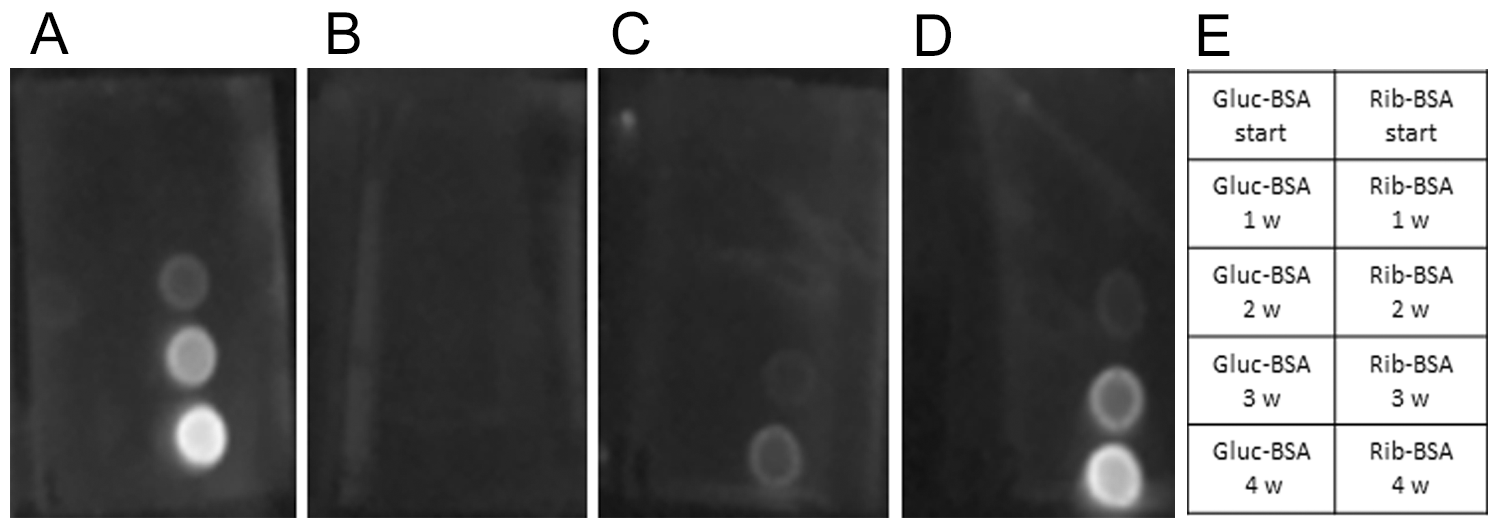

Supplement: S4 Fig — (A) D1-B2, (B) negative control (no antibody), (C) KH011, (D) KH025, (E) antigen positions on dotblot. (TIF) [file pone.0191872.s004.tif]

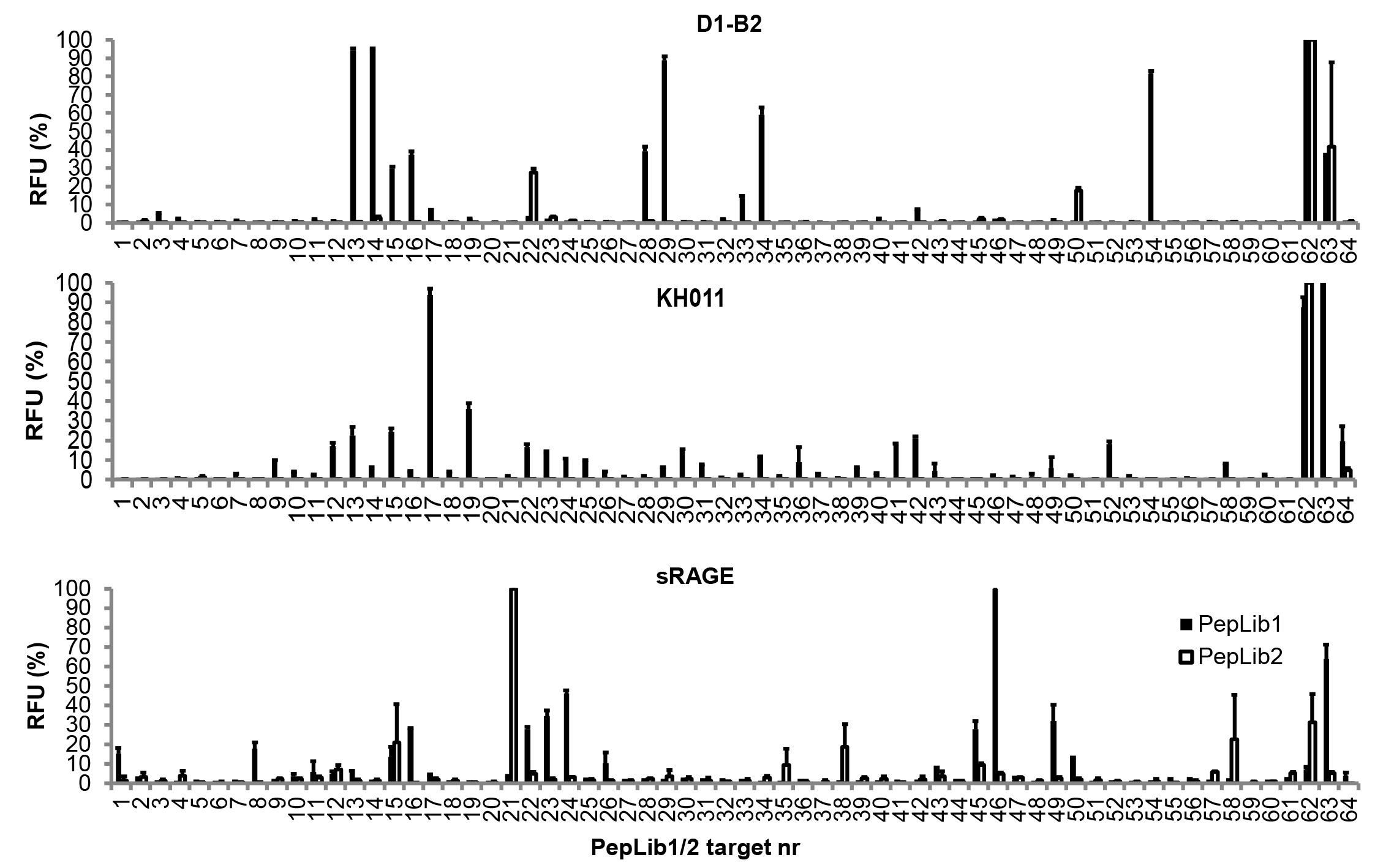

Supplement: S5 Fig — Normalized signals in % were calculated by dividing each RFU value with the maximal RFU value in the same analysis and then multiplying with 100. (TIF) [file pone.0191872.s005.tif]
