# Supplementary material for: A novel monoclonal antibody targeting carboxymethyllysine, an advanced glycation end product in atherosclerosis and pancreatic cancer
Source: PLoS One. 2018 Feb 8;13(2):e0191872. doi: 10.1371/journal.pone.0191872 (PMC5805250; doi:10.1371/journal.pone.0191872)
Supplement: S1 Table — (PDF) [file pone.0191872.s006.pdf]

S1 Table: CML-BSA peptide library PepLib1

Peptide nr	20mer aa stretch	Amino acid sequence
1	1-20	MK*WVTFISLLLLFSSAYSRG
2	11-30	LLFSSAYSRGVFRRDTHK*SE
3	21-40	VFRRDTHK*SEIAHRFK*DLGE
4	31-50	IAHRFK*DLGEEHFK*GLVLIA
5	41-60	EHFK*GLVLIAFSQYLQQCPF
6	51-70	FSQYLQQCPFDEHVK*LVNEL
7	61-80	DEHVK*LVNELTEFAK*TCVAD
8	71-90	TEFAK*TCVADESHAGCEK*SL
9	81-100	ESHAGCEK*SLHTLFGDELCK*
10	91-110	HTLFGDELCK*VASLRETYGD
11	101-120	VASLRETYGDMADCCEK*QEP
12	111-130	MADCCEK*QEPERNECFLSHK*
13	121-140	ERNECFLSHK*DDSPDLPK*LK*
14	131-150	DDSPDLPK*LK*PDPNTLCDEF
15	141-160	PDPNTLCDEFK*ADEK*K*FWGK*
16	151-170	K*ADEK*K*FWGK*YLYEIARRHP
17	161-180	YLYEIARRHPYFYAPELLYY
18	171-190	YFYAPELLYYANK*YNGVFQE
19	181-200	ANK*YNGVFQECCQAEDK*GAC
20	191-210	CCQAEDK*GACLLPK*IETMRE
21	201-220	LLPK*IETMREK*VLASSARQR
22	211-230	K*VLASSARQRLRCASIQK*FG
23	221-240	LRCASIQK*FGERALK*AWSVA
24	231-250	ERALK*AWSVARLSQK*FPK*AE
25	241-260	RLSQK*FPK*AEFVEVTK*LVTD
26	251-270	FVEVTK*LVTDLTK*VHK*ECCH
27	261-280	LTK*VHK*ECCHGDLLECADDR
28	271-290	GDLLECADDRADLAK*YICDN
29	281-300	ADLAK*YICDNQDTISSK*LK*E
30	291-310	QDTISSK*LK*ECCDK*PLLEK*S
31	301-320	CCDK*PLLEK*SHCIAEVEK*DA
32	311-330	HCIAEVEK*DAIPENLPPLTA
33	321-340	IPENLPPLTADFAEDK*DVCK*
34	331-350	DFAEDK*DVCK*NYQEAK*DAFL
35	341-360	NYQEAK*DAFLGSFLYEYSRR
36	351-370	GSFLYEYSRRHPEYAVSVLL
37	361-380	HPEYAVSVLLRLAK*EYEATL
38	371-390	RLAK*EYEATLEECCAK*DDPH
39	381-400	EECCAK*DDPHACYSTVFDK*L
40	391-410	ACYSTVFDK*LK*HLVDEPQNL
41	401-420	K*HLVDEPQNLIK*QNCDQFEK*
42	411-430	IK*QNCDQFEK*LGEYGFQNAL
43	421-440	LGEYGFQNALIVRYTRK*VPQ
44	431-450	IVRYTRK*VPQVSTPTLVEVS
45	441-460	VSTPTLVEVSRSLGK*VGTRC
46	451-470	RSLGK*VGTRCCTK*PESERMP
47	461-480	CTK*PESERMPCTEDYLSLIL
48	471-490	CTEDYLSLILNRLCVLHEK*T

49	481-500	NRLCVLHEK*TPVSEK*VTK*CC
50	491-510	PVSEK*VTK*CCTESLVNRRPC
51	501-520	TESLVNRRPCFSALTPDETY
52	511-530	FSALTPDETYVPK*AFDEK*LF
53	521-540	VPK*AFDEK*LFTFHADICTLP
54	531-550	TFHADICTLPDTEK*QIK*K*QT
55	541-560	DTEK*QIK*K*QTALVELLK*HK*P
56	551-570	ALVELLK*HK*PK*ATEEQLK*TV
57	561-580	K*ATEEQLK*TVMENFVAFVDK*
58	571-590	MENFVAFVDK*CCAADDK*EAC
59	581-600	CCAADDK*EACFAVEGPK*LVV
60	591-607	FAVEGPK*LVVSTQTALA
61		unmodified BSA (negative control)
62		CML-BSA, reductive amination (positive control)
63		CML-IgG, reductive amination (positive control)
64		Peptide 1-6 (positive controls)
