# Supplementary material for: A novel monoclonal antibody targeting carboxymethyllysine, an advanced glycation end product in atherosclerosis and pancreatic cancer
Source: PLoS One. 2018 Feb 8;13(2):e0191872. doi: 10.1371/journal.pone.0191872 (PMC5805250; doi:10.1371/journal.pone.0191872)
Supplement: S2 Table — (PDF) [file pone.0191872.s007.pdf]

S2 Table: BSA peptide library PepLib2

Peptide nr	20mer aa stretch	Amino acid sequence
1	1-20	MKWVTFISLLLLFSSAYSRG
2	11-30	LLFSSAYSRGVFRRDTHKSE
3	21-40	VFRRDTHKSEIAHRFKDLGE
4	31-50	IAHRFKDLGEEHFKGLVLIA
5	41-60	EHFKGLVLIAFSQYLQQCPF
6	51-70	FSQYLQQCPFDEHVKLVNEL
7	61-80	DEHVKLVNELTEFAKTCVAD
8	71-90	TEFAKTCVADESHAGCEKSL
9	81-100	ESHAGCEKSLHTLFGDELCK
10	91-110	HTLFGDELCKVASLRETYGD
11	101-120	VASLRETYGDMADCCEKQEP
12	111-130	MADCCEKQEPERNECFLSHK
13	121-140	ERNECFLSHKDDSPDLPKLK
14	131-150	DDSPDLPKLKPDPNTLCDEF
15	141-160	PDPNTLCDEFKADEKKFWGK
16	151-170	KADEKKFWGKYLYEIARRHP
17	161-180	YLYEIARRHPYFYAPELLYY
18	171-190	YFYAPELLYYANKYNGVFQE
19	181-200	ANKYNGVFQECCQAEDKGAC
20	191-210	CCQAEDKGACLLPKIETMRE
21	201-220	LLPKIETMREKVLASSARQR
22	211-230	KVLASSARQRLRCASIQKFG
23	221-240	LRCASIQKFGERALKAWSVA
24	231-250	ERALKAWSVARLSQKFPKAE
25	241-260	RLSQKFPKAEFVEVTKLVTD
26	251-270	FVEVTKLVTDLTKVHKECCH
27	261-280	LTKVHKECCHGDLLECADDR
28	271-290	GDLLECADDRADLAKYICDN
29	281-300	ADLAKYICDNQDTISSKLKE
30	291-310	QDTISSKLKECCDKPLLEKS
31	301-320	CCDKPLLEKSHCIAEVEKDA
32	311-330	HCIAEVEKDAIPENLPPLTA
33	321-340	IPENLPPLTADFAEDKDVCK
34	331-350	DFAEDKDVCKNYQEAKDAFL
35	341-360	NYQEAKDAFLGSFLYEYSRR
36	351-370	GSFLYEYSRRHPEYAVSVLL
37	361-380	HPEYAVSVLLRLAKEYEATL
38	371-390	RLAKEYEATLEECCAKDDPH
39	381-400	EECCAKDDPHACYSTVFDKL
40	391-410	ACYSTVFDKLKHLVDEPQNL
41	401-420	KHLVDEPQNLIKQNCDQFEK
42	411-430	IKQNCDQFEKLGEYGFQNAL
43	421-440	LGEYGFQNALIVRYTRKVPQ
44	431-450	IVRYTRKVPQVSTPTLVEVS
45	441-460	VSTPTLVEVSRSLGKVGTRC
46	451-470	RSLGKVGTRCCTKPESERMP
47	461-480	CTKPESERMPCTEDYLSLIL
48	471-490	CTEDYLSLILNRLCVLHEKT

49	481-500	NRLCVLHEKTPVSEKVTKCC
50	491-510	PVSEKVTKCCTESLVNRRPC
51	501-520	TESLVNRRPCFSALTPDETY
52	511-530	FSALTPDETYVPKAFDEKLF
53	521-540	VPKAFDEKLFTFHADICTLP
54	531-550	TFHADICTLPDTEKQIKKQT
55	541-560	DTEKQIKKQTALVELLKHKP
56	551-570	ALVELLKHKPKATEEQLKTV
57	561-580	KATEEQLKTVMENFVAFVDK
58	571-590	MENFVAFVDKCCAADDKEAC
59	581-600	CCAADDKEACFAVEGPKLVV
60	591-607	FAVEGPKLVVSTQTALA
61		unmodified BSA (negative control)
62		CML-BSA, reductive amination (positive control)
63		CML-IgG, reductive amination (positive control)
64		Peptide 1-6 (positive controls)
