# Supplementary material for: A novel monoclonal antibody targeting carboxymethyllysine, an advanced glycation end product in atherosclerosis and pancreatic cancer
Source: PLoS One. 2018 Feb 8;13(2):e0191872. doi: 10.1371/journal.pone.0191872 (PMC5805250; doi:10.1371/journal.pone.0191872)
Supplement: S3 Table — (PDF) [file pone.0191872.s008.pdf]

S3 Table: Epitope mapping library PepLib3

Peptide nr	Amino acid sequence
1	DDSPDLP(CML)L(CML)PDPNTLCDEF
2	ADSPDLP(CML)L(CML)PDPNTLCDEF
3	DASPDLP(CML)L(CML)PDPNTLCDEF
4	DDAPDLP(CML)L(CML)PDPNTLCDEF
5	DDSADLP(CML)L(CML)PDPNTLCDEF
6	DDSPALP(CML)L(CML)PDPNTLCDEF
7	DDSPDAP(CML)L(CML)PDPNTLCDEF
8	DDSPDLA(CML)L(CML)PDPNTLCDEF
9	DDSPDLPAL(CML)PDPNTLCDEF
10	DDSPDLP(CML)A(CML)PDPNTLCDEF
11	DDSPDLP(CML)LAPDPNTLCDEF
12	DDSPDLP(CML)L(CML)ADPNTLCDEF
13	DDSPDLP(CML)L(CML)PAPNTLCDEF
14	LP(CML)L(CML)PDP
15	PDLP(CML)L(CML)PDPN
16	DPLP(CML)L(CML)PDPN
17	PLDP(CML)L(CML)PDPN
18	PLPD(CML)L(CML)PDPN
19	PDLP(CML)L(CML)DPPN
20	PDLP(CML)L(CML)PPDN
21	PDLP(CML)L(CML)PPND
22	PLPD(CML)L(CML)DPPN
23	PLDP(CML)L(CML)PDPN
24	PDLP(CML)L(CML)PPDN
25	DPLP(CML)L(CML)PPND
26	PELP(CML)L(CML)PDPN
27	PDLP(CML)L(CML)PEPN
28	PELP(CML)L(CML)PEPN
29	PDLP(CML)A(CML)PDPN
30	PDLP(CML)C(CML)PDPN
31	PDLP(CML)D(CML)PDPN
32	PDLP(CML)E(CML)PDPN
33	PDLP(CML)F(CML)PDPN
34	PDLP(CML)G(CML)PDPN
35	PDLP(CML)H(CML)PDPN
36	PDLP(CML)I(CML)PDPN
37	PDLP(CML)K(CML)PDPN
38	PDLP(CML)L(CML)PDPN
39	PDLP(CML)M(CML)PDPN
40	PDLP(CML)N(CML)PDPN
41	PDLP(CML)P(CML)PDPN
42	PDLP(CML)Q(CML)PDPN
43	PDLP(CML)R(CML)PDPN
44	PDLP(CML)S(CML)PDPN
45	PDLP(CML)T(CML)PDPN
46	PDLP(CML)V(CML)PDPN
47	PDLP(CML)W(CML)PDPN
48	PDLP(CML)Y(CML)PDPN

49	DDSPDLP(CML)L(CML)PDANTLCDEF
50	DDSPDLP(CML)L(CML)PDPATLCDEF
51	DDSPDLP(CML)L(CML)PDPNALCDEF
52	DDSPDLP(CML)L(CML)PDPNTACDEF
53	DDSPDLP(CML)L(CML)PDPNTLADEF
54	DDSPDLP(CML)L(CML)PDPNTLCAEF
55	DDSPDLP(CML)L(CML)PDPNTLCDAF
56	DSPDLP(CML)L(CML)PDPNTLCDE
57	SPDLP(CML)L(CML)PDPNTLCD
58	PDLP(CML)L(CML)PDPNTLC
59	DLP(CML)L(CML)PDPNTL
60	P(CML)L(CML)PDPN
61	P(CML)L(CML)PDP
62	P(CML)L(CML)PD
63	P(CML)L(CML)P
64	(CML)L(CML)
65	DDSPDLP(SL)L(SL)PDPNTLCDEF
66	CML-BSA reductive amination (positive control)
67	BSA (negative control)
68	CML-IgG, reductive amination (positive control)
69	IgG (negative control)
