# Supplementary material for: A novel monoclonal antibody targeting carboxymethyllysine, an advanced glycation end product in atherosclerosis and pancreatic cancer
Source: PLoS One. 2018 Feb 8;13(2):e0191872. doi: 10.1371/journal.pone.0191872 (PMC5805250; doi:10.1371/journal.pone.0191872)
Supplement: S4 Table — (PDF) [file pone.0191872.s009.pdf]

S4 Table: Glycated protein library ProtLib1

Target nr	Protein	Protein concentration (mg/mL)	Carbohydrate	Carbohydrate concentration (mM)	pH	T (°C)	Incubation time
1	BSA	50	glucose	500	7.2	37	1 day
2	BSA	50	glucose	500	7.2	37	2 days
3	BSA	50	glucose	500	7.2	37	3 days
4	BSA	50	glucose	500	7.2	37	1 week
5	BSA	50	glucose	500	7.2	37	2 weeks
6	BSA	50	glucose	500	7.2	37	3 weeks
7	BSA	50	glucose	500	7.2	37	4 weeks
8	BSA	50	glucose	500	10	37	1 day
9	BSA	50	glucose	500	10	37	2 days
10	BSA	50	glucose	500	10	37	3 days
11	BSA	50	glucose	500	10	37	1 week
12	BSA	50	glucose	500	10	37	2 weeks
13	BSA	50	glucose	500	10	37	3 weeks
14	BSA	50	glucose	500	10	37	4 weeks
15	BSA	50	glucose	500	7.2	50	1 day
16	BSA	50	glucose	500	7.2	50	2 days
17	BSA	50	glucose	500	7.2	50	3 days
18	BSA	50	glucose	500	7.2	50	1 week
19	BSA	50	glucose	500	7.2	50	2 weeks
20	BSA	50	glucose	500	7.2	50	3 weeks
21	BSA	50	glucose	500	7.2	50	4 weeks
22	BSA	50	ribose	500	7.2	37	1 day
23	BSA	50	ribose	500	7.2	37	2 days
24	BSA	50	ribose	500	7.2	37	3 days
25	BSA	50	ribose	500	7.2	37	1 week
26	BSA	50	ribose	500	7.2	37	2 weeks
27	BSA	50	ribose	500	7.2	37	3 weeks
28	BSA	50	ribose	500	7.2	37	4 weeks
29	BSA	50	fructose	500	7.2	37	1 day
30	BSA	50	fructose	500	7.2	37	2 days
31	BSA	50	fructose	500	7.2	37	3 days

32	BSA	50	fructose	500	7.2	37	1 week
33	BSA	50	fructose	500	7.2	37	2 weeks
34	BSA	50	fructose	500	7.2	37	3 weeks
35	BSA	50	fructose	500	7.2	37	4 weeks
36	BSA	50	Glyceraldehyde	100	7.2	37	2 weeks
37	BSA	50	Methylglyoxal	100	7.2	37	2 weeks
38	BSA	50	glyoxal	100	7.2	37	2 weeks
39	BSA	50	-	-	-	-	-
40	HSA	50	glucose	500	7.2	37	4 weeks
41	HSA	50	ribose	500	7.2	37	4 weeks
42	HSA	50	fructose	500	7.2	37	4 weeks
43	HSA	50	Glyceraldehyde	100	7.2	37	2 weeks
44	HSA	50	Methylglyoxal	100	7.2	37	2 weeks
45	HSA	50	glyoxal	100	7.2	37	2 weeks
46	HSA	50	-	-	-	-	-
47	IgG	10	glucose	500	7.2	37	4 weeks
48	IgG	10	ribose	500	7.2	37	4 weeks
49	IgG	10	fructose	500	7.2	37	4 weeks
50	IgG	10	Glyceraldehyde	100	7.2	37	2 weeks
51	IgG	10	Methylglyoxal	100	7.2	37	2 weeks
52	IgG	10	glyoxal	100	7.2	37	2 weeks
53	IgG	10	-	-	-	-	-
54	Fibrinogen	10	glucose	500	7.2	37	4 weeks
55	Fibrinogen	10	ribose	500	7.2	37	4 weeks
56	Fibrinogen	10	fructose	500	7.2	37	4 weeks
57	Fibrinogen	10	Glyceraldehyde	100	7.2	37	2 weeks
58	Fibrinogen	10	Methylglyoxal	100	7.2	37	2 weeks
59	Fibrinogen	10	glyoxal	100	7.2	37	2 weeks
60	Fibrinogen	10	-	-	-	-	-
61	Collagen	0.1	glucose	500	7.2	37	4 weeks
62	Collagen	0.1	ribose	500	7.2	37	4 weeks
63	Collagen	0.1	fructose	500	7.2	37	4 weeks
64	Collagen	0.1	Glyceraldehyde	100	7.2	37	2 weeks

65	Collagen	0.1	Methylglyoxal	100	7.2	37	2 weeks
66	Collagen	0.1	glyoxal	100	7.2	37	2 weeks
67	Collagen	0.1	-	-	-	-	-
68	Collagen	0.25	glucose	500	7.2	37	4 weeks
69	Collagen	0.25	ribose	500	7.2	37	4 weeks
70	Collagen	0.25	fructose	500	7.2	37	4 weeks
71	Collagen	0.25	Glyceraldehyde	100	7.2	37	2 weeks
72	Collagen	0.25	Methylglyoxal	100	7.2	37	2 weeks
73	Collagen	0.25	glyoxal	100	7.2	37	2 weeks
74	Collagen	0.25	-	-	-	-	-
