# Supplementary material for: A novel monoclonal antibody targeting carboxymethyllysine, an advanced glycation end product in atherosclerosis and pancreatic cancer
Source: PLoS One. 2018 Feb 8;13(2):e0191872. doi: 10.1371/journal.pone.0191872 (PMC5805250; doi:10.1371/journal.pone.0191872)
Supplement: S5 Table — (PDF) [file pone.0191872.s010.pdf]

S5 Table: Binding of D1-B2 to peptide library PepLib1

Peptide nr	Amino acid sequence	RFU	Stdev
1	M(CML)WVTFISLLLLFSSAYSRG	131	22
2	LLFSSAYSRGVFRRDTH(CML)SE	44	16
3	VFRRDTH(CML)SEIAHRF(CML)DLGE	3137	154
4	IAHRF(CML)DLGEEHF(CML)GLVLIA	1505	97
5	EHF(CML)GLVLIAFSQYLQQCPF	305	54
6	FSQYLQQCPFDEHV(CML)LVNEL	296	32
7	DEHV(CML)LVNELTEFA(CML)TCVAD	711	68
8	TEFA(CML)TCVADESHAGCE(CML)SL	144	19
9	ESHAGCE(CML)SLHTLFGDELC(CML)	210	49
10	HTLFGDELC(CML)VASLRETYGD	529	67
11	VASLRETYGDMADCCE(CML)QEP	1257	41
12	MADCCE(CML)QEPERNECFLSH(CML)	545	4
13	ERNECFLSH(CML)DDSPDLP(CML)L(CML)	59529	840
14	DDSPDLP(CML)L(CML)PDPNTLCDEF	59843	579
15	PDPNTLCDEF(CML)ADE(CML)(CML)FWG(CML)	18739	777
16	(CML)ADE(CML)(CML)FWG(CML)YLYEIARRHP	23564	1121
17	YLYEIARRHPYFYAPELLYY	3704	726
18	YFYAPELLYYAN(CML)YNGVFQE	321	53
19	AN(CML)YNGVFQECCQAED(CML)GAC	1442	57
20	CCQAED(CML)GACLLP(CML)IETMRE	-8	3
21	LLP(CML)IETMRE(CML)VLASSARQR	129	39
22	(CML)VLASSARQRLRCASIQ(CML)FG	1596	229
23	LRCASIQ(CML)FGERAL(CML)AWSVA	828	58
24	ERAL(CML)AWSVARLSQ(CML)FP(CML)AE	148	26
25	RLSQ(CML)FP(CML)AEFVEVT(CML)LVTD	328	33
26	FVEVT(CML)LVTDLT(CML)VH(CML)ECCH	444	17
27	LT(CML)VH(CML)ECCHGDLLECADDR	91	46
28	GDLLECADDRADLA(CML)YICDN	24609	1642
29	ADLA(CML)YICDNQDTISS(CML)L(CML)E	56151	1410
30	QDTISS(CML)L(CML)ECCD(CML)PLLE(CML)S	397	71
31	CCD(CML)PLLE(CML)SHCIAEVE(CML)DA	364	23
32	HCIAEVE(CML)DAIPENLPPLTA	1193	27
33	IPENLPPLTADFAED(CML)DVC(CML)	8557	845
34	DFAED(CML)DVC(CML)NYQEA(CML)DAFL	37325	2651
35	NYQEA(CML)DAFLGSFLYEYSRR	40	25
36	GSFLYEYSRRHPEYAVSVLL	108	41
37	HPEYAVSVLLRLA(CML)EYEATL	50	16
38	RLA(CML)EYEATLEECCA(CML)DDPH	139	6
39	EECCA(CML)DDPHACYSTVFD(CML)L	215	24
40	ACYSTVFD(CML)L(CML)HLVDEPQNL	1522	26
41	(CML)HLVDEPQNLI(CML)QNCDQFE(CML)	172	28
42	I(CML)QNCDQFE(CML)LGEYGFQNAL	4512	252
43	LGEYGFQNALIVRYTR(CML)VPQ	152	46
44	IVRYTR(CML)VPQVSTPTLVEVS	73	14
45	VSTPTLVEVSRSLG(CML)VGTRC	199	31
46	RSLG(CML)VGTRCCT(CML)PESERMP	679	79
47	CT(CML)PESERMPCTEDYLSLIL	73	20
48	CTEDYLSLILNRLCVLHE(CML)T	70	27

49	NRLCVLHE(CML)TPVSE(CML)VT(CML)CC	806	268
50	PVSE(CML)VT(CML)CCTESLVNRRPC	135	43
51	TESLVNRRPCFSALTPDETY	91	27
52	FSALTPDETYVP(CML)AFDE(CML)LF	179	13
53	VP(CML)AFDE(CML)LFTFHADICTLP	335	116
54	TFHADICTLPDTE(CML)QI(CML)(CML)QT	51656	967
55	DTE(CML)QI(CML)(CML)QTALVELL(CML)H(CML)P	6	20
56	ALVELL(CML)H(CML)P(CML)ATEEQL(CML)TV	48	10
57	(CML)ATEEQL(CML)TVMENFVAFVD(CML)	232	213
58	MENFVAFVD(CML)CCAADD(CML)EAC	36	15
59	CCAADD(CML)EACFAVEGP(CML)LVV	61	6
60	FAVEGP(CML)LVVSTQTALA	196	29
61	unmodified BSA (negative control)	84	
62	CML-BSA, reductive amination (positive control)	63225	
63	CML-IgG reductive amination, (positive control)	23008	
64	Peptide 1-6 (positive controls)	-4	
