# Supplementary material for: A novel monoclonal antibody targeting carboxymethyllysine, an advanced glycation end product in atherosclerosis and pancreatic cancer
Source: PLoS One. 2018 Feb 8;13(2):e0191872. doi: 10.1371/journal.pone.0191872 (PMC5805250; doi:10.1371/journal.pone.0191872)
Supplement: S6 Table — (PDF) [file pone.0191872.s011.pdf]

S6 Table: Binding of D1-B2 to peptide library PepLib2

Peptide nr	Amino acid sequence	RFU	Stdev
1	MKWVTFISLLLLFSSAYSRG	15	2
2	LLFSSAYSRGVFRRDTHKSE	483	306
3	VFRRDTHKSEIAHRFKDLGE	11	16
4	IAHRFKDLGEEHFKGLVLIA	40	25
5	EHFKGLVLIAFSQYLQQCPF	71	13
6	FSQYLQQCPFDEHVKLVNEL	83	23
7	DEHVKLVNELTEFAKTCVAD	38	26
8	TEFAKTCVADESHAGCEKSL	38	39
9	ESHAGCEKSLHTLFGDELCK	82	11
10	HTLFGDELCKVASLRETYGD	100	11
11	VASLRETYGDMADCCEKQEP	13	8
12	MADCCEKQEPERNECFLSHK	88	11
13	ERNECFLSHKDDSPDLPKLK	253	45
14	1DDSPDLPKLKPDPNTLCDEF	1288	248
15	PDPNTLCDEFKADEKKFWGK	122	38
16	KADEKKFWGKYLYEIARRHP	223	25
17	YLYEIARRHPYFYAPELLYY	17	12
18	YFYAPELLYYANKYNGVFQE	22	22
19	ANKYNGVFQECCQAEDKGAC	86	32
20	CCQAEDKGACLLPKIETMRE	48	8
21	LLPKIETMREKVLASSARQR	22	46
22	KVLASSARQRLRCASIQKFG	12411	982
23	LRCASIQKFGERALKAWSVA	1478	104
24	ERALKAWSVARLSQKFPKAE	546	32
25	RLSQKFPKAEFVEVTKLVTD	26	16
26	FVEVTKLVTDLTKVHKECCH	30	32
27	LTKVHKECCHGDLLECADDR	136	24
28	GDLLECADDRADLAKYICDN	494	9
29	ADLAKYICDNQDTISSKLKE	67	16
30	QDTISSKLKECCDKPLLEKS	80	27
31	CCDKPLLEKSHCIAEVEKDA	4	4
32	HCIAEVEKDAIPENLPPLTA	47	21
33	IPENLPPLTADFAEDKDVCK	4	16
34	DFAEDKDVCKNYQEAKDAFL	17	24
35	NYQEAKDAFLGSFLYEYSRR	7	8
36	GSFLYEYSRRHPEYAVSVLL	121	81
37	HPEYAVSVLLRLAKEYEATL	-4	3
38	RLAKEYEATLEECCAKDDPH	89	15
39	EECCAKDDPHACYSTVFDKL	65	14
40	ACYSTVFDKLKHLVDEPQNL	0	27
41	KHLVDEPQNLIKQNCDQFEK	63	5
42	IKQNCDQFEKLGEYGFQNAL	47	4
43	LGEYGFQNALIVRYTRKVPQ	414	25
44	IVRYTRKVPQVSTPTLVEVS	116	41
45	VSTPTLVEVSRSLGKVGTRC	986	204
46	RSLGKVGTRCCTKPESERMP	766	214
47	CTKPESERMPCTEDYLSLIL	36	26
48	CTEDYLSLILNRLCVLHEKT	14	4

49	NRLCVLHEKTPVSEKVTKCC	37	27
50	PVSEKVTKCCTESLVNRRPC	8074	615
51	TESLVNRRPCFSALTPDETY	41	9
52	FSALTPDETYVPKAFDEKLF	-5	15
53	VPKAFDEKLFTFHADICTLP	2	11
54	TFHADICTLPDTEKQIKKQT	44	17
55	DTEKQIKKQTALVELLKHKP	27	7
56	ALVELLKHKPKATEEQLKTV	77	44
57	KATEEQLKTVMENFVAFVDK	19	19
58	MENFVAFVDKCCAADDKEAC	218	90
59	CCAADDKEACFAVEGPKLVV	104	36
60	FAVEGPKLVVSTQTALA	15	3
61	unmodified BSA (negative control)	28	
62	CML-BSA, reductive amination (positive control)	45350	
63	CML-IgG reductive amination, (positive control)	18897	
64	Peptide 1-6 (positive controls)	330	
