# Supplementary material for: A novel monoclonal antibody targeting carboxymethyllysine, an advanced glycation end product in atherosclerosis and pancreatic cancer
Source: PLoS One. 2018 Feb 8;13(2):e0191872. doi: 10.1371/journal.pone.0191872 (PMC5805250; doi:10.1371/journal.pone.0191872)
Supplement: S7 Table — (PDF) [file pone.0191872.s012.pdf]

S7 Table: Binding of D1-B2 to peptide library PepLib3

Peptide nr	Amino acid sequence	RFU	Stdev
1	DDSPDLP(CML)L(CML)PDPNTLCDEF	30042	11855
2	ADSPDLP(CML)L(CML)PDPNTLCDEF	56414	1112
3	DASPDLP(CML)L(CML)PDPNTLCDEF	47876	4857
4	DDAPDLP(CML)L(CML)PDPNTLCDEF	49550	1076
5	DDSADLP(CML)L(CML)PDPNTLCDEF	57762	1056
6	DDSPALP(CML)L(CML)PDPNTLCDEF	6926	1116
7	DDSPDAP(CML)L(CML)PDPNTLCDEF	54840	608
8	DDSPDLA(CML)L(CML)PDPNTLCDEF	51637	1600
9	DDSPDLPAL(CML)PDPNTLCDEF	2612	297
10	DDSPDLP(CML)A(CML)PDPNTLCDEF	61738	419
11	DDSPDLP(CML)LAPDPNTLCDEF	60168	581
12	DDSPDLP(CML)L(CML)ADPNTLCDEF	51099	2372
13	DDSPDLP(CML)L(CML)PAPNTLCDEF	59487	377
14	LP(CML)L(CML)PDP	121	115
15	PDLP(CML)L(CML)PDPN	20529	353
16	DPLP(CML)L(CML)PDPN	688	90
17	PLDP(CML)L(CML)PDPN	737	92
18	PLPD(CML)L(CML)PDPN	501	32
19	PDLP(CML)L(CML)DPPN	21593	1188
20	PDLP(CML)L(CML)PPDN	46557	798
21	PDLP(CML)L(CML)PPND	6007	105
22	PLPD(CML)L(CML)DPPN	207	35
23	PLDP(CML)L(CML)PDPN	28	1
24	PDLP(CML)L(CML)PPDN	48201	455
25	DPLP(CML)L(CML)PPND	3270	598
26	PELP(CML)L(CML)PDPN	1338	156
27	PDLP(CML)L(CML)PEPN	13599	630
28	PELP(CML)L(CML)PEPN	391	44
29	PDLP(CML)A(CML)PDPN	20114	1128
30	PDLP(CML)C(CML)PDPN	51597	2291
31	PDLP(CML)D(CML)PDPN	20802	349
32	PDLP(CML)E(CML)PDPN	53449	373
33	PDLP(CML)F(CML)PDPN	53018	924
34	PDLP(CML)G(CML)PDPN	30667	1588
35	PDLP(CML)H(CML)PDPN	31201	32
36	PDLP(CML)I(CML)PDPN	53748	313
37	PDLP(CML)K(CML)PDPN	47701	1722
38	PDLP(CML)L(CML)PDPN	37306	1871
39	PDLP(CML)M(CML)PDPN	13406	741
40	PDLP(CML)N(CML)PDPN	14097	430
41	PDLP(CML)P(CML)PDPN	23624	203
42	PDLP(CML)Q(CML)PDPN	20492	1400
43	PDLP(CML)R(CML)PDPN	4	22
44	PDLP(CML)S(CML)PDPN	35401	2300
45	PDLP(CML)T(CML)PDPN	43528	1413
46	PDLP(CML)V(CML)PDPN	51308	3198
47	PDLP(CML)W(CML)PDPN	55166	793
48	PDLP(CML)Y(CML)PDPN	57157	530

49	DDSPDLP(CML)L(CML)PDANTLCDEF	44210	754
50	DDSPDLP(CML)L(CML)PDPATLCDEF	44558	197
51	DDSPDLP(CML)L(CML)PDPNALCDEF	32132	1087
52	DDSPDLP(CML)L(CML)PDPNTACDEF	21562	1538
53	DDSPDLP(CML)L(CML)PDPNTLADEF	44990	1375
54	DDSPDLP(CML)L(CML)PDPNTLCAEF	11624	882
55	DDSPDLP(CML)L(CML)PDPNTLCDAF	50981	1175
56	DSPDLP(CML)L(CML)PDPNTLCDE	50500	3261
57	SPDLP(CML)L(CML)PDPNTLCD	59204	579
58	PDLP(CML)L(CML)PDPNTLC	55390	563
59	DLP(CML)L(CML)PDPNTL	50525	1309
60	P(CML)L(CML)PDPN	3256	687
61	P(CML)L(CML)PDP	4718	774
62	P(CML)L(CML)PD	719	133
63	P(CML)L(CML)P	1389	147
64	(CML)L(CML)	2559	455
65	DDSPDLP(SL)L(SL)PDPNTLCDEF	-4	15
66	CML-BSA reductive amination (positive control)	35221	184
67	BSA (negative control)	40	48
68	CML-IgG, reductive amination (positive control)	17779	614
69	IgG (negative control)	20058	261
