# Supplementary material for: A novel monoclonal antibody targeting carboxymethyllysine, an advanced glycation end product in atherosclerosis and pancreatic cancer
Source: PLoS One. 2018 Feb 8;13(2):e0191872. doi: 10.1371/journal.pone.0191872 (PMC5805250; doi:10.1371/journal.pone.0191872)
Supplement: S8 Table — (PDF) [file pone.0191872.s013.pdf]

S8 Table: Binding of D1-B2 to glycated protein library ProtLib1

Target nr/description	RFU	Stdev
1. BSA, glucose, pH 7.2, 37°C, 1 d	938	140
2. BSA, glucose, pH 7.2, 37°C, 2 d	2411	877
3. BSA, glucose, pH 7.2, 37°C, 3 d	1125	336
4. BSA, glucose, pH 7.2, 37°C, 1 w	2924	513
5. BSA, glucose, pH 7.2, 37°C, 2 w	1215	178
6. BSA, glucose, pH 7.2, 37°C, 3 w	871	501
7. BSA, glucose, pH 7.2, 37°C, 4 w	193	213
8. BSA, glucose, pH 10, 37°C, 1 d	110	134
9. BSA, glucose, pH 10, 37°C, 2 d	27	11
10. BSA, glucose, pH 10, 37°C, 3 d	191	84
11. BSA, glucose, pH 10, 37°C, 1 w	1196	135
12. BSA, glucose, pH 10, 37°C, 2 w	2795	24
13. BSA, glucose, pH 10, 37°C, 3 w	3270	34
14. BSA, glucose, pH 10, 37°C, 4 w	7174	615
15. BSA, glucose, pH 7.2, 50°C, 1 d	-20	17
16. BSA, glucose, pH 7.2, 50°C, 2 d	1264	573
17. BSA, glucose, pH 7.2, 50°C, 3 d	500	63
18. BSA, glucose, pH 7.2, 50°C, 1 w	144	81
19. BSA, glucose, pH 7.2, 50°C, 2 w	18	20
20. BSA, glucose, pH 7.2, 50°C, 3 w	70	49
21. BSA, glucose, pH 7.2, 50°C, 4 w	308	64
22. BSA, ribose, pH 7.2, 37°C, 1 d	-12	20
23. BSA, ribose, pH 7.2, 37°C, 2 d	71	16
24. BSA, ribose, pH 7.2, 37°C, 3 d	666	29
25. BSA, ribose, pH 7.2, 37°C, 1 w	2658	70
26. BSA, ribose, pH 7.2, 37°C, 2 w	7852	140
27. BSA, ribose, pH 7.2, 37°C, 3 w	13364	585
28. BSA, ribose, pH 7.2, 37°C, 4 w	16595	721
29. BSA, fructose, pH 7.2, 37°C, 1 d	735	211
30. BSA, fructose, pH 7.2, 37°C, 2 d	73	59
31. BSA, fructose, pH 7.2, 37°C, 3 d	16	38
32. BSA, fructose, pH 7.2, 37°C, 1 w	456	358
33. BSA, fructose, pH 7.2, 37°C, 2 w	1262	672
34. BSA, fructose, pH 7.2, 37°C, 3 w	735	492
35. BSA, fructose, pH 7.2, 37°C, 4 w	587	333
36. BSA, glyceraldehyde, pH 7.2, 37°C, 2 w	1259	32
37. BSA, methylglyoxal, pH 7.2, 37°C, 2 w	4436	668
38. BSA, glyoxal, pH 7.2, 37°C, 2 w	62501	264
39. BSA, unmodified	1815	1235
40. HSA, glucose, pH 7.2, 37°C, 4 w	367	565
41. HSA, ribose, pH 7.2, 37°C, 4 w	2543	177
42. HSA, fructose, pH 7.2, 37°C, 4 w	79	96
43. HSA, glyceraldehyde, pH 7.2, 37°C, 2 w	97	6
44. HSA, methylglyoxal, pH 7.2, 37°C, 2 w	65	9
45. HSA, glyoxal, pH 7.2, 37°C, 2 w	5909	855
46. HSA, unmodified	-120	33
47. IgG, glucose, pH 7.2, 37°C, 4 w	389	67
48. IgG, ribose, pH 7.2, 37°C, 4 w	1318	67

49. IgG, fructose, pH 7.2, 37°C, 4 w	227	75
50. IgG, glyceraldehyde, pH 7.2, 37°C, 2 w	6008	441
51. IgG, methylglyoxal, pH 7.2, 37°C, 2 w	4366	227
52. IgG, glyoxal, pH 7.2, 37°C, 2 w	1808	348
53. IgG, unmodified	215	43
54. Fibrinogen, glucose, pH 7.2, 37°C, 4 w	380	93
55. Fibrinogen, ribose, pH 7.2, 37°C, 4 w	647	72
56. Fibrinogen, fructose, pH 7.2, 37°C, 4 w	3454	358
57. Fibrinogen, glyceraldehyde, pH 7.2, 37°C, 2 w	1028	69
58. Fibrinogen, methylglyoxal, pH 7.2, 37°C, 2 w	7280	285
59. Fibrinogen, glyoxal, pH 7.2, 37°C, 2 w	9839	233
60. Fibrinogen, unmodified	1243	522
61. 0.1 mg/ml collagen, glucose, pH 7.2, 37°C, 4 w	50	33
62. 0.1 mg/ml collagen, ribose, pH 7.2, 37°C, 4 w	56	2
63. 0.1 mg/ml collagen, fructose, pH 7.2, 37°C, 4 w	52	15
64. 0.1 mg/ml collagen, glyceraldehyde, pH 7.2, 37°C, 2 w	118	10
65. 0.1 mg/ml collagen, methylglyoxal, pH 7.2, 37°C, 2 w	278	56
66. 0.1 mg/ml collagen, glyoxal, pH 7.2, 37°C, 2 w	267	83
67. 0.1 mg/ml collagen, unmodified	-31	39
68. 0.25 mg/ml collagen, glucose, pH 7.2, 37°C, 4 w	88	52
69. 0.25 mg/ml collagen, ribose, pH 7.2, 37°C, 4 w	110	22
70. 0.25 mg/ml collagen, fructose, pH 7.2, 37°C, 4 w	173	109
71. 0.25 mg/ml collagen, glyceraldehyde, pH 7.2, 37°C, 2 w	628	44
72. 0.25 mg/ml collagen, methylglyoxal, pH 7.2, 37°C, 2 w	246	33
73. 0.25 mg/ml collagen, glyoxal, pH 7.2, 37°C, 2 w	206	32
74. 0.25 mg/ml collagen, unmodified	624	349
